# Cost-effectiveness of pharmacological therapies for people with Alzheimer’s disease and other dementias: a systematic review and meta-analysis

**DOI:** 10.1186/s12962-022-00354-3

**Published:** 2022-04-20

**Authors:** Zhaohua Huo, Jiaer Lin, Baker K. K. Bat, Tak Kit Chan, Benjamin H. K. Yip, Kelvin K. F. Tsoi

**Affiliations:** 1 JC School of Public Health and Primary Care, Prince of Wales Hospital, The Chinese University of Hong Kong, Hong Kong, China; 2 Stanley Ho Big Data Decision Analytics Research Centre, The Chinese University of Hong Kong, Hong Kong, China

**Keywords:** Cost, Cost-effectiveness, Dementia, Drug, Acetylcholinesterase inhibitors, Memantine

## Abstract

**Objectives:**

This study aims to synthesize the empirical economic evidence of pharmaceutical therapies for people with dementia.

**Study design:**

Systematic review and meta-analysis. Literature evaluating the costs and effects of drug therapies for dementia was indexed until December 2021. Quality of study was assessed using the Cochrane Risk of Bias Tool and Consensus on Health Economic Criteria list. Cost data were standardized to 2020 US dollars and analyzed from healthcare service and societal perspectives. Random-effects models were used to synthesize economic and clinical data, based on mean differences (MDs) and standardized MDs.

**Results:**

Ten unique studies were identified from 11,771 records. Acetylcholinesterase inhibitors (AChEIs) and memantine improved dementia-related symptoms, alongside nonsignificant savings in societal cost (AChEIs: MD-2002 [− 4944 ~ 939]; memantine: MD-6322 [− 14355 ~ 1711]). Despite decreases in cost, antidepressants of mirtazapine and sertraline and second-generation antipsychotics were limited by their significant side effects on patients’ cognitive and activity functions. Subgroup analysis indicated that the impacts of AChEIs on cost were affected by different analytical perspectives, follow-up periods, and participant age.

**Conclusions:**

AChEIs and memantine are cost-effective with improvements in dementia-related symptoms and trends of cost-savings. More empirical evidence with non-industrial sponsorships and rigorous design in different settings is warranted.

**Supplementary Information:**

The online version contains supplementary material available at 10.1186/s12962-022-00354-3.

## Background

Dementia is affecting over 47 million people worldwide, and it brings an economic burden of over US$1 trillion per year [1, 2]. Because of limited therapies, acetylcholinesterase inhibitors (AChEIs) and memantine are still the first choices for treating dementia for now, especially for Alzheimer’s disease (AD) [3]. The monotherapy or combined use of them have been proved effective in improving dementia-related symptoms in different stages of AD or other dementias, including cognitive, activity and global functioning [4–6]. In terms of neuropsychiatric symptoms related to dementia, the use of antipsychotics and antidepressants was proposed, but raising concerns through the side effects induced meanwhile [5, 6]. The prescription of this kind of drugs should consider both the benefits and harms after regular assessment and discussion with patients and their family members [3, 7].

Besides the clinical benefit of different medications for dementia, the cost-effectiveness of them is also a crucial issue for decision and policy making, especially when confronting the imbalance of growing healthcare needs and constrained resources in an aging society [8, 9]. Given that the pharmacological treatments for AD and other dementias can be costly, one matter for consideration is whether the direct cost of these drugs can be eclipsed by the cost savings owing to the remission of caregiving workload or delayed institutionalization of patients. Previous studies indicated that drug therapies for AD were more effective and less costly than placebo or no treatments [9]. However, recommendations for clinical practice and expense reimbursement were still prudent [9]. One reason for this is most supportive evidence came from modeling studies, where the validity of results could be influenced by the variations and ambiguities in study design, models, and data input [9, 10]. High-quality and empirical evidence from randomized controlled trials (RCTs) and prospective cohort studies is scarce [11]. In addition, existing economic evidence has deficiencies in the outcome measurements, sponsorships mainly from industrial entities, and investigations in medications other than AChEIs and memantine [9]. Quantitative synthesis is also lacking on it. In order to provide a clearer synthesis on existing evidence and prospect for future studies in this area, this study aims to appraise the empirical evidence of the cost-effectiveness of different pharmacological interventions for Alzheimer’s disease and other dementias, and to perform meta-analyses on the costs and effects of them.

## Methods

This review followed the Preferred Reporting Items for a Systematic Review and Meta-analysis (PRISMA) [12], Centre for Reviews and Dissemination [13] and Cochrane Handbook [14]. The PRISMA checklist is provided in Additional file 1: Appendix 1.

### Search strategy

Literature was searched from PubMed, Web of Science, EMBASE, Cochrane Library Database, Science Direct, SCOPUS, PsychoINFO, CINAHL, EconLit, NHS Economic Evaluation Database and three Chinese databases (Chinese Biomedical Literature Database, WANGFANG, CNKI), from their inceptions to December 2021. Grey literature was retrieved from the OpenSIGLE website and the early detection and timely INTERvention in DEMentia website. Medical Subject Headings and natural terms related to “dementia”, “pharmacological” and “economic evaluation” were used in the search strategies, and the search results are presented (Additional file 1: Tables A1, A2 in Appendix 2). Manual search was extended to the reference lists of relevant reviews and included studies until no new records were identified.

### Study selection

The PICOS criteria are shown in Additional file 1: Appendix 3. Full (i.e., cost-effectiveness, cost–benefit, cost-utility, cost-minimization analysis) and partial economic evaluations (i.e., cost-consequence analysis) were both eligible. Trial-based study were prioritized, and quasi-experimental and observational studies were also eligible. Modelling-based studies were excluded due to large discrepancies in models used, assumptions, data sources, and data inputs, according to previous findings [9]. In sum, studies were included if they evaluated any pharmacological interventions for AD or other dementias; targeted at people with any type or any stage of dementia in any setting; involved a control group treated with placebo, no treatment, or other alternatives; and reported the costs and health outcomes of people with dementia. Studies were excluded if they were modelling designs; involved diseases out of the scope of this study; evaluated preventive interventions; were qualitative, cost-of-illness, methodological, or review articles; only measured cost of studied medications; or were published in languages other than English and Chinese.

### Data extraction

Information extracted included the study design, participants recruitment and characteristics, interventions and comparators, measures and results of costs and health outcomes, incremental cost-effectiveness ratios (ICERs), analytical perspective, sensitivity analysis, and quality of study. Cost data were compiled based on different perspectives and re-categorized into three types: intervention cost, healthcare utilization cost, and indirect cost of productivity loss or informal care [2, 15]. Total costs only included cost of intervention and healthcare utilization from healthcare system perspectives, while from societal perspectives, they additionally included cost of informal care and indirect cost [8, 15]. To reduce information bias, any publications on the study design and clinical outcomes linked to the included studies were retrieved. Results on repeatedly measured health outcomes were prioritized if they matched the cost data, otherwise, those with longer follow-ups were chosen.

### Quality assessment

The quality of RCTs and non-randomized trials was assessed by the revised Cochrane risk-of-bias tool [14, 16] and Risk of Bias in Non-randomized studies of Interventions tool [16], respectively. The methodological quality of economic evaluation was assessed using the 19-item Consensus on Health Economic Criteria list [17].

Two investigators (Z.H, J.L) independently screened the records for eligibility. Data were extracted by one investigator (Z.H) and double checked by another investigator (J.L). Quality of study was also assessed independently by the two same investigators. Any disagreements were first discussed by the two reviewers, and if unresolved, referred to the study team for consensus.

### Statistical analysis

The primary outcomes of interest were the incremental cost and effects of interventions relative to the controls. Heterogeneity across studies was examined through Cochran’s Q tests, with a cut-off P-level of 0.10. Publication bias was investigated through funnel plots and Egger's tests [14]. Quantitative synthesis was performed using random-effects meta-analysis models, based on mean differences (MDs) and standardized mean differences (SMDs) [14]. The Cohen’s d effect size of SMDs could be regarded as small (0.2), medium (0.5) or large (0.8) [18]. Health outcomes were categorized into five domains: cognition, activity functioning, global deterioration, behavioral and psychological symptoms of dementia (BPSDs), and health related quality of life (HRQoL). Different scales measuring the same domain in the single study were re-calculated into a composite measure, using the average SMD of these scales [19]. Cost data were synthesized from two perspectives: healthcare system perspectives and societal perspectives. Monetary unit was standardized to US dollar (September 2020) using the CCEMG-EPPICentre Cost Converter (v.1.6) [20]. Cost-effectiveness (C-E) planes were used to visualize the relative cost and effects of interventions compared to controls [21].

Potential factors associated with the costs and effects were investigated in subgroup analyses, including participant characteristics, follow-up period, and social context. Univariate meta-regression was performed if there were enough studies. One-way sensitivity analysis was performed, where only RCT-based studies, only industry-sponsored studies, and only self-rating HRQoL scales were included in the analysis. Sensitivity analysis was also performed for economic evaluations that calculated cost data as a function of cognitive or activity scores based on clinical trials. Quantitative analyses were performed using the Meta procedures in STATA 16 (StataCorp, Tx) [22]. P-value at significant levels was set at 0.05 except for additional specifications.

## Results

### Study selection

A total of 11,585 records were identified. The titles and abstracts of 10,616 unique records were screened, and 10,472 of them were excluded mainly due to irrelevance. The full text of 144 articles were retrieved. Of these, 134 were excluded due to duplications, inappropriate types of study, modeling studies, insufficient data, or other reasons. Ten publications based on ten unique studies were finally included (Additional file 1: Fig. A1 in Appendix 3).

### Study characteristics

#### Study design, intervention, and context

The characteristics of the ten included studies are summarized (Additional file 1: Tables A4, A5 in Appendix 4). Eight studies investigated drugs treating the cognitive decline and global deterioration of dementia (i.e., AChEIs, memantine and propentofylline), and the other two studies investigated psychotropics treating the depressive and agitation symptoms related to dementia. All studies selected placebo or no treatment as controls, and three studies investigated more than one drug. All studies recruited participants from community settings, and three also recruited participants from residential homes. Nine of the ten studies were RCTs, conducted in European or North American countries, and lasting for one year or less. Seven studies were funded or donated by industrial entities.

#### Target population

A total of 3664 participants were involved, and the median sample size was 311. All study samples had a mean age between 70 and 79 years. Nine studies recruited participants with AD or vascular dementia, and one study recruited participants with Parkinson’s disease dementia. Five studies investigated mild to moderate stages of dementia, while three studied moderate-to-severe dementia.

#### Clinical and economic evaluation

Measures of health outcomes included cognitive functions (n = 9), activity of daily living (n = 10), global deterioration (n = 7), BPSDs (n = 7), and HRQoL or quality-adjusted life years (QALYs) gained (n = 4); only one study investigated participants’ destination of institutionalization. Six studies employed full economic evaluations: two were cost-effectiveness analyses, and four performed both cost-effectiveness and cost-utility analyses. For cost collection, five studies adopted societal perspectives, two adopted healthcare service or payer perspectives, and three adopted both. In addition, three studies measured the healthcare utilization of informal caregivers.

### Quality assessment

Among nine RCTs, five showed a low risk of bias, while three showed some concerns, and one showed high risk of bias (Additional file 1: Table A6 and Fig. A2 in Appendix S5). The domain with largest concerns was handling of missing outcome data. The risk of bias of the single quasi-experimental study was high due to the inappropriate selection of participants, handling of missing data, and outcome measurements. The methodological quality of economic evaluation is also presented (Additional file 1: Table A7 and Fig. A3 in Appendix 5). Eight of ten studies met at least 85% of quality criteria; however, only one met all criteria. Items with the lowest fulfillments were “no penitential conflict of interest” and “incremental analysis performed”.

### Data synthesis

Extracted cost and clinical outcomes are compiled in Additional file 1: Tables A8, A9 (Appendix 6). The incremental cost per participant of each comparison is presented (Table 1). Regardless of the perspective undertaken, 9 of 16 comparisons (56.3%) have shown net savings in total cost, including AChEIs (n = 3), and memantine (n = 2), mirtazapine (n = 2), and risperidone (n = 1). Quantitative synthesis was based on different types of drugs. Heterogeneity across studies was only detected in the effects of AChEIs (Additional file 1: Table A10 in Appendix 6). These variations mainly caused by Knapp (2017)’s study overlapped with the potential small-study effects detected (Additional file 1: Fig. A4 in Appendix 6). We first performed meta-analyses based on different types of drugs, and then explored the potential source of heterogeneity through subgroup and sensitivity analyses.

**Table 1:**
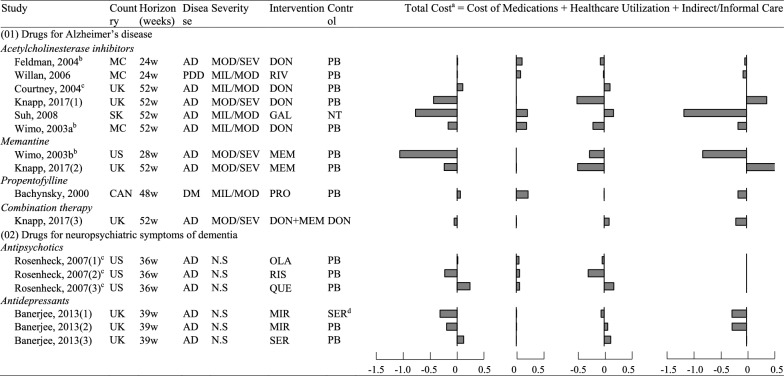
Incremental cost of pharmacological interventions for people with dementia (per participant or per dyad, 10,000 USD, 2020 price year)

^a^Cost collection was based on societal perspectives in all included studies, except for Courtney (2004)’s and Rosenheck (2007)’s studies. Negative values indicated cost savings in the intervention group compared to the control group

^b^Cost collection included health utilization of informal caregivers

^c^Cost collection was only based on health care perspectives and did not consider indirect cost or informal care

^d^Data were not included into quantitative synthesis due to alternative drugs as comparators

AD: Alzheimer’s Disease; CAN: Canada; DM: Dementia; DON: Donepezil; GAL:Galantamine; MC: multi-centres; MEM: Memantine; MIL: Mild; MIR: Mirtazapine; MOD: Moderate; N.S: Not specified; NT: no treatment; OLA: Olanzapine; PB: Placebo; PDD: Parkinson Disease Dementia; PRO: Propentofylline; RIS: Risperidone; QUE: Quetiapine; RIV: Rivastigmine; SER: Sertraline; SEV: Severe; SK: South Korea; UK: United Kingdom; US: United States

#### Medications for AD and other dementias

Eight studies with 2917 participants studied drugs for AD and other dementias: three in donepezil [23–25], and the other five respectively in galantamine [26], rivastigmine [27], memantine [28], propentofylline [29], and the combined use of donepezil and memantine [30]. The C-E planes based on individual studies indicated that these medications were mostly effective as well as cost-saving from societal perspectives (Additional file 1: Fig. A5 (1) in Appendix 6). Based on the results of meta-analysis, acetylcholinesterase inhibitors increased the healthcare cost (MD: 859, 95% CI [− 150, 1847]); however, when taking informal care into consideration, there was an inverse trend of cost savings in total cost (MD: − 2002 [− 4944, 939]) (Table 2). They also showed significant effects on patients’ cognition (SMD: 0.561 [0.277, 0.846], P < 0.001), activity functioning (SMD: 0.450 [0.240, 0.661], P < 0.001), global deterioration (SMD: 0.306 [0.181, 0.432], P < 0.001), and BPSDs (SMD: 0.238 [0.052, 0.424], P < 0.001) (Table 3). The cost-effectiveness of memantine for patients with dementia was more apparent, indicating cost savings from both healthcare and societal perspectives, as well as significant improvements in cognitive and activity functions (Tables 2, 3). Compared to monotherapy, the economic evidence of combination therapy was less explicit due to the limited amount of study. As for ICERs for QALYs gained, only two studies reported the results, and they concluded the acceptance of donepezil, memantine and rivastigmine when compared to placebo, with ICER values ranging from cost-savings to CAN$7429 [27, 30]. However, the combined use of donepezil and memantine was not cost-effective compared to using donepezil alone due to too high ICER value (> £30,000) [30].

**Table 2:** Meta-analysis on the incremental cost of pharmacological interventions for people with dementia

Type of intervention	Incremental total cost (Intervention group–Control group) ^a^			
	n	Healthcare perspective	n	Societal perspective
(01) Drugs for Alzheimer’s disease				
Acetylcholinesterase inhibitors	5	MD: 859 (− 150, 1847) SMD: 0.090 (− 0.016, 0.197)	5	MD: -2002 (− 4944, 939) SMD: − 0.109 (− 0.283, 0.064)
Memantine	1^b^	MD: − 2283 (− 7874, 3309) SMD: − 0.213 (− 0.736, 0.310)	2	MD: -6322 (− 14,355, 1711) SMD: − 0.328 (− 0.594, − 0.063)^*^
Propentofylline	1^b^	MD: 2171 (1085, 3256) SMD: 0.340 (0.169, 0.512)	0	–
Combination therapy	1^b^	MD: 970 (− 3568, 5508) SMD: 0.106 (− 0.389, 0.600)	1^b^	MD: − 536 (− 7426, 6353) SMD: − 0.038 (− 0.532, 0.455)
(02) Drugs for neuropsychiatric symptoms of dementia				
Antipsychotics	3	MD: − 574 (− 7141, 5993) SMD: − 0.008 (− 0.211, 0.195)	0	–
Antidepressants	2	MD: 684 (− 1648, 3015) SMD: 0.091 (− 0.222, 0.404)	2	MD: − 660 (− 4620, 3301) SMD: − 0.041 (− 0.354, 0.272)

^a^Negative values based on mean differences (MD) or standardized mean differences (SMD) indicated cost savings in the intervention group compared to the control group

^b^No meta-analysis was performed due to insufficient number of studies, and the result was derived from a single study

*P-value < 0.05; **P-value < 0.01; ***, P-value < 0.001

**Table 3:** Meta-analysis on the effects of pharmacological interventions for people with dementia

Type of intervention	Effects on health outcomes (Intervention group–Control group) ^a^											
	n	Cognition	n	Activity functions	n	Global deterioration	n	BPSD	n	Health utility	n	QALY
(01) Drugs for Alzheimer’s disease												
Acetylcholinesterase inhibitors	6^c^	0.561^***^ (0.277, 0.846)	6^c^	0.450^***^ (0.240, 0.661)	3	0.306^***^ (0.181, 0.432)	4^c^	0.238^*^ (0.052, 0.424)	1^b^	− 0.323 (− 0.781, 0.136)	2^c^	0.341 (− 0.136, 0.818)
Memantine	2	0.602^***^ (0.290, 0.914)	2	0.445^***^ (0.208, 0.682)	1^b^	0.337 (0.043, 0.631)	1^b^	0.618 (0.217, 1.019)	1^b^	0.224 (− 0.170, 0.618)	1^b^	0.327 (− 0.068, 0.772)
Propentofylline	1^b^	0.228 (0.057, 0.398)	1^b^	0.212 (0.041, 0.382)	1^b^	0.207 (0.036, 0.377)	0	–	0	–	0	–
Combination therapy	1^b^	0.376 (− 0.022, 0.775)	1^b^	0.118 (− 0.278, 0.513)	0	–	1^b^	0.568 (0.166, 0.971)	1^b^	0.249 (− 0.148, 0.646)	1^b^	0.092 (− 0.303, 0.488)
(02) Drugs for neuropsychiatric symptoms of dementia												
Antipsychotics	0	–	3	− 0.192^*^ (− 0.369, − 0.014)	3	0.189 (− 0.014, 0.392)	0	–	3	− 0.022 (− 0.225, 0.181)	3	0.020 (− 0.183, 0.223)
Antidepressants	2	− 0.383^*^ (− 0.762, − 0.003)	2	− 0.242 (− 0.560, 0.076)	0	–	2	0.035 (− 0.278, 0.347)	2	0.006 (− .368, 0.379)	2	0.378^*^ (0.002, 0.754)

^a^Positive values indicated better improvements in the health domain in the intervention group compared to the control group

^b^Meta-synthesis was not performed due to insufficient number of studies, and the result was derived from a single study

^c^Heterogeneity was detected across studies based on Cochran's Q test (P < 0.10)

*P-value < 0.05; **P-value < 0.01; ***P-value < 0.001

#### Medications for neuropsychiatric symptoms of dementia

Two RCT-based studies with 747 participants investigated drugs treating neuropsychiatric symptoms of dementia. One study evaluated the second generation of antipsychotics (i.e., olanzapine, risperidone, and quetiapine) [31] and the other investigated antidepressants (i.e., mirtazapine and sertraline) [32]. The C-E planes based on individual studies did not show an intuitive trend of changes in the costs of these types of medications; however, there was a tendency of deterioration in cognitive and activity functions using these drugs for patients with dementia (Additional file 1: Fig. A5 (2) in Appendix 6). The pooled estimates showed that antipsychotics were associated with lower healthcare cost (MD: − 574 [− 7141, 5993]) while antidepressants decreased societal cost (MD: − 660 [− 4620, 3301]); however, these differences were nonsignificant (Table 2). Regarding health benefits, antipsychotics and antidepressants only showed limited improvements in patients’ neuropsychiatric symptoms; by contrast, they were associated with significant deterioration in patients’ cognition (antidepressants: SMD − 0.383 [− 0.762, − 0.003], P = 0.048) or activity functions (antipsychotics: SMD − 0.192 [− 0.369, − 0.014]; P = 0.034) of participants (Table 3). The only health benefits were the improvements of antidepressants in QALYs gained (SMD: 0.378 [0.002, 0.754], P = 0.049). As for ICERs for QALYs gained, both studies reported the results. Only mirtazapine compared to sertraline or placebo was concluded to be cost-effective with better outcomes and lower cost [32]. Other medications (sertraline, olanzapine, risperidone, and quetiapine) were rejected by judgments of too high ICER values beyond the acceptable thresholds [32, 33].

### Subgroup analysis

#### Study perspective

Five studies reported cost information from both healthcare and societal perspectives. After considering informal care among AChEIs, the direction of incremental cost of drug interventions altered from positive to negative, and the number decreased at a significant level (difference in MD: − 3172, P = 0.025) (Additional file 1: Table A11 in Appendix 7). However, inclusion of caregivers’ healthcare utilization did not have significant impacts on the incremental cost (Additional file 1: Table A12 in Appendix 7).

#### Follow-up period

Among six studies of AChEIs, two involved a time horizon of 24 weeks, while the other four involved around 52 weeks. The incremental healthcare cost and effects of AChEIs did not differ significantly by different follow-up periods in subgroup analysis. Nevertheless, when taking informal care into consideration, use of AChEIs in longer follow-up periods was associated with significantly lower societal cost (difference in MD: − 4847, P = 0.028) (Additional file 1: Table A13 in Appendix 7).

#### Disease severity and age

The baseline characteristics of participants also affected the cost-effectiveness of AChEIs. Subgroup analysis showed that AChEIs used for moderate-to-severe dementia (versus mild-to-moderate dementia) were more effective in BPSD symptoms (difference in SMD: 0.292, P = 0.036), while these drugs used among older patients (mean age ≥ 75 versus < 75 years) were more cost-saving from societal perspectives (difference in MD: − 6342, P = 0.010) (Additional file 1: Table A13 in Appendix 7).

#### Social context

Multinational studies indicated smaller effects of AChEIs on the incremental societal cost and health outcomes compared to single-cite studies. Nevertheless, no significant differences were found (Additional file 1: Table A13 in Appendix 7).

### Sensitivity analysis

**Fig. 1:**
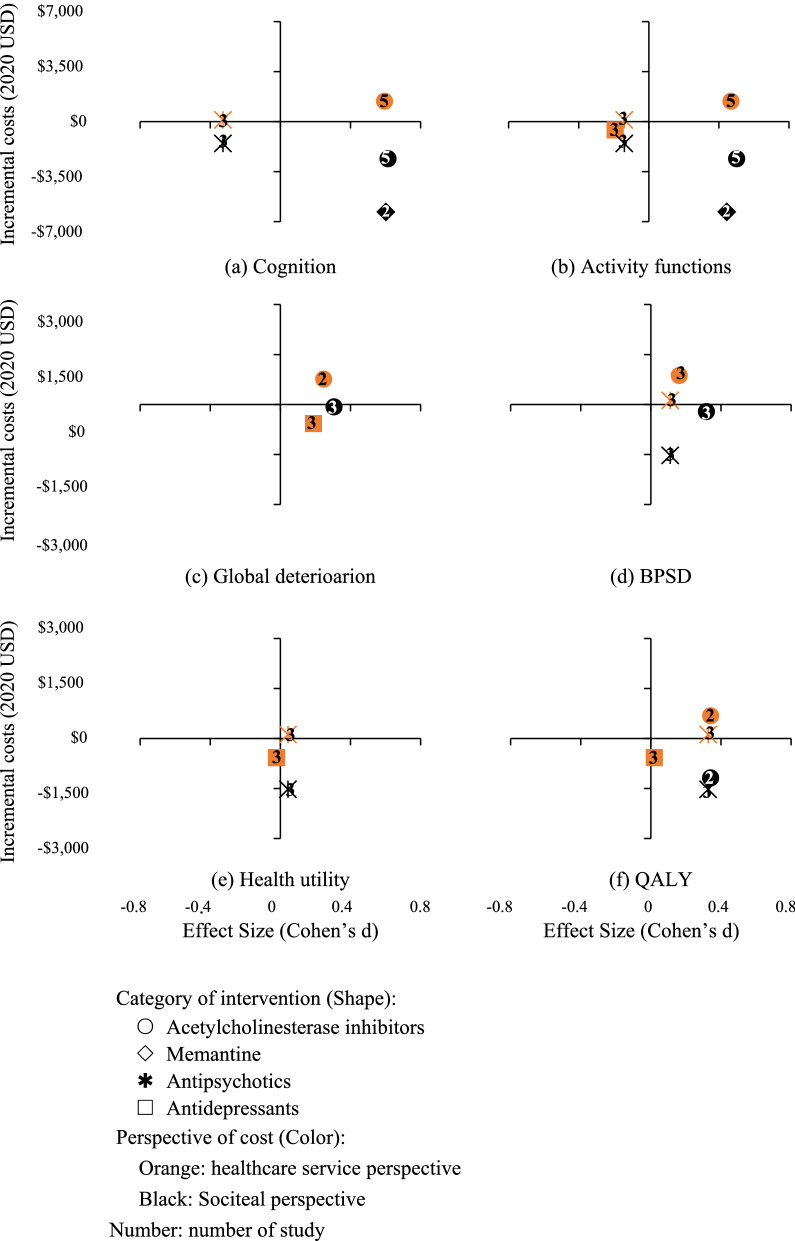
Cost-effectiveness planes based on pooled estimates of studies with complete data on costs and effects in the sensitivity analysis

Firstly, cost data were analyzed based on both MDs and SMDs (Table 2). The only difference was the savings of memantine in societal cost calculated by SMDs turned to be significant. We eventually chose to report cost based on MDs because they were more intuitive, and the overall conclusions did not change between using MDs and SMDs. In sensitivity analyses that only included RCT-based studies, and that only considered industry-sponsored studies, no significant differences were found compared to the primary analysis (Additional file 1: Tables A14, A15 in Appendix 8). However, in the sensitivity analysis that compared the self-rated and proxy-rated scales, the effects of antidepressants on patients’ HRQoL showed large differences (self-rated vs proxy-rated scales: SMD − 0.146 vs 0.159) (Additional file 1: Table A16 in Appendix 8). In the sensitivity analysis that only considered studies with data on both costs and effects in specific domains, the overall findings did not change (Additional file 1: Table A17 in Appendix 8), and the C-E planes based on the pooled results also conformed to the primary findings (Fig. 1). Finally, the incremental societal cost of AChEIs remained consistent after including the study that calculated cost data as a function of activity functioning score (MD: − 2293, 95% CI [− 4983, 397]) [34].

## Discussion

### Main findings

This study reviewed and synthesized the economic evidence of different medications for people with dementia from ten empirical and trial-based studies. Acetylcholinesterase inhibitors and memantine for AD and other dementias were found cost-effective in improving dementia-related symptoms alongside nonsignificant savings in societal cost. These findings were consistent with previous studies [9, 35]. We also reviewed the cost-effectiveness of psychotropic drugs for individuals with dementia. Although antidepressants indicated higher QALYs for patients alongside neutral incremental cost, their negative impacts on patients’ cognitive functions should not be neglected. They were also limited by benefits on neuropsychiatric symptoms. Meanwhile, antipsychotics were not regarded as cost-effective due to limited benefits on healthcare cost and health outcomes, alongside significant side effects on activity functions. Subgroup analyses indicated that AChEIs were more cost-saving after considering informal care, usage in a longer period (≥ 1 year) and among older persons (mean age ≥ 75 years) with dementia; meanwhile, they were more effective among persons with moderate-to-severe dementia.

### Strengths and limitations

To our knowledge, this is the first study to perform quantitative synthesis on the economic evidence of pharmacological therapies for persons with dementia. The search of evidence was restricted to empirical studies to avoid the uncertainty and ambiguity underlying modeling studies. Secondly, to gain a comprehensive view in both clinical and economic aspects, any additional information regarding the clinical effects of interventions was retrieved and included in analyses. Composite measures were used to summarize the overall effects of interventions on different domains of health outcomes, and these were gradually accepted in systematic reviews and data syntheses [36, 37]. Thirdly, for cost data, we synthesized data using random-effect models based on both MD and SMD to test the robustness of results. Subgroup analyses and sensitivity analyses were also performed to investigate the potential sources of heterogeneity that could influence the results.

This study, however, possesses some limitations. Firstly, the validity of data synthesis was impaired by the heterogeneity detected, especially in AChEIs. Although these variations could partly be explained by differences in participant characteristics (the study as the main source of heterogeneity also had severer and older participants [30]), the number of studies was still too few to preclude other possibilities, such as non-reporting biases (i.e., economic evaluations could be more likely performed in interventions with positive clinical effects) or chance. Secondly, only two of the ten included studies were sponsored by non-industrial entities. Potential bias introduced by industry-sponsorships was hard to preclude, despite no significant exaggerations being found in the sensitivity analysis. Thirdly, the generalizability of findings could be restricted because most evidence was derived from high-income countries, community-dwelling participants with AD and vascular dementia, and short follow-up periods. Most of included evidence was also dated, and the innovation, manufacturing and price of drugs and the routine care for dementia may change over time and influence the costs and effects of dementia drugs. Fourthly, collection and presentation of cost data varied across studies. Some studies did not measure informal care, and some did not provide sufficient cost information (e.g., variances of cost data). Handling of missing data was also inexplicit in four of the ten included studies. These led to incomplete data for analysis and could bring potential risk of bias. Finally, we found inconsistency in the impacts of specific intervention on HRQoL scales and QALYs derived. This may be explained by the health utility scales used to calibrate QALYs differing from those reported in the original study [30, 32]. The selection of self-rating or proxy-rating scales could also influence the value of health utility and QALYs [14, 38], which was also reflected in our sensitivity analysis.

### Implications

For clinical practice, our findings support the routine use of AChEIs and memantine for patients with dementia. However, antipsychotics or antidepressants for patients with dementia should be prescribed with caution in the limited benefits on clinical and economic outcomes, accompanied by the significant side effects. For policy making, with aging populations and increasing new drugs approved for treating AD (e.g., aducanumab), the demands and related expense of dementia-related drugs will be predictably expanding. The clinical and cost-effectiveness of different dementia drugs requires re-scrutiny before updating recommendations or compensation policies. Although our results revealed the cost-effectiveness of AChEIs and memantine for treating dementia, the validity could be diluted by unexplored sources of heterogeneity and most studies sponsored by industrial entities. These should be considered in decision making. For future studies in this area, since current AD drugs have become parts of clinical guideline and placebo-control can be ethically prohibitive, more focus can be turned towards other innovative drugs (e.g., disease-modifying medications), combined use of different drugs for severer dementia, combination of drug and non-drug therapies, and head-to-head comparisons between different medications. For example, the US Food and Drug Administration approved a new amyloid beta-directed antibody aducanumab (Aduhelm; BiogenInc) for treating AD recently in July 2021 [39]. The cost-effectiveness of aducanumab compared to current standard care can depend largely on the pricing of drug and requires intensive research [40]. Study designs of further studies should also be improved with non-industrial sponsorships, long-term follow-ups, reliable measurements in HRQoL of patients, and cost collection from both healthcare and societal perspectives. Evidence from residential care settings and developing and non-western countries is needed as well.

## Conclusions

Acetylcholinesterase inhibitors and memantine were found cost-effective with significant improvements in patients’ dementia-related symptoms, alongside nonsignificant savings in total societal cost. Antipsychotic and antidepressant drugs for patient with dementia had less impacts on cost; and they were limited by the side effects on patients’ cognitive or activity functions. Heterogeneity in the incremental cost and effects of AChEIs could partly be explained by different analytical perspectives, follow-up periods and baseline characteristics of participants. Future empirical studies independent from industrial entities were warranted on the cost-effectiveness of innovative drugs, combined use of different drugs, long-term impacts of interventions, and drug use in residential care settings and developing countries.

## Supplementary Information

**Additional file 1.** Supplementary contents.

## Data Availability

All data generated or analyzed during this study are included in this published article and its supplementary information files.

## Abbreviations

AChEI: Acetylcholinesterase inhibitor
AD: Alzheimer’s disease
BPSD: Behavioral and psychological symptom of dementia
C-E: Cost-effectiveness
HRQoL: Health related quality of life
ICER: Incremental cost-effectiveness ratio
MD: Mean difference
PRISMA: Preferred Reporting Items for a Systematic Review and Meta-analysis
RCT: Randomized controlled trial
SMD: Standardized mean difference
