# Supplementary material for: Cost-effectiveness of pharmacological therapies for people with Alzheimer’s disease and other dementias: a systematic review and meta-analysis
Source: Cost Eff Resour Alloc. 2022 Apr 20;20:19. doi: 10.1186/s12962-022-00354-3 (PMC9022294; doi:10.1186/s12962-022-00354-3)
Supplement: Supplementary file 1 — Additional file 1. Supplementary contents. [file 12962_2022_354_MOESM1_ESM.docx]

**Supplementary Contents**

[Appendix 1: PRISMA checklist 1](#_Toc66803425)

[Appendix 2: Search strategies and results 4](#_Toc66803426)

[Appendix 3: PICOS criteria and study selection 9](#_Toc66803427)

[Appendix 4: Characteristics of eligible studies 11](#_Toc66803428)

[Appendix 5: Quality assessment 17](#_Toc66803429)

[Appendix 6: Costs and effects of interventions 22](#_Toc66803430)

[Appendix 7: Subgroup analysis 32](#_Toc66803431)

[Appendix 8: Sensitivity analysis 37](#_Toc66803432)

# Appendix 1: PRISMA checklist

| Section/topic | # | Checklist item | Reported on page # |
| --- | --- | --- | --- |
| **TITLE** | | | |
| Title | 1 | Identify the report as a systematic review, meta-analysis, or both. | Page 1 (Title) |
| **ABSTRACT** | | | |
| Structured summary | 2 | Provide a structured summary including, as applicable: background; objectives; data sources; study eligibility criteria, participants, and interventions; study appraisal and synthesis methods; results; limitations; conclusions and implications of key findings; systematic review registration number. | Page 2 (Abstract) |
| **INTRODUCTION** | | | |
| Rationale | 3 | Describe the rationale for the review in the context of what is already known. | Page 3-4 (Background) |
| Objectives | 4 | Provide an explicit statement of questions being addressed with reference to participants, interventions, comparisons, outcomes, and study design (PICOS). |  |
| **METHODS** | | | |
| Protocol and registration | 5 | Indicate if a review protocol exists, if and where it can be accessed (e.g., Web address), and, if available, provide registration information including registration number. | NA |
| Eligibility criteria | 6 | Specify study characteristics (e.g., PICOS, length of follow-up) and report characteristics (e.g., years considered, language, publication status) used as criteria for eligibility, giving rationale. | Page 4-5 (Study selection), Appendix 3 (PICOS) |
| Information sources | 7 | Describe all information sources (e.g., databases with dates of coverage, contact with study authors to identify additional studies) in the search and date last searched. | Page 4 (Search Strategy) |
| Search | 8 | Present full electronic search strategy for at least one database, including any limits used, such that it could be repeated. | Appendix 2 (Search strategies and results) |
| Study selection | 9 | State the process for selecting studies (i.e., screening, eligibility, included in systematic review, and, if applicable, included in the meta-analysis). | Page 4-5 (Study selection) |
| Data collection process | 10 | Describe method of data extraction from reports (e.g., piloted forms, independently, in duplicate) and any processes for obtaining and confirming data from investigators. | Page 4-5 (Data extraction) |
| Data items | 11 | List and define all variables for which data were sought (e.g., PICOS, funding sources) and any assumptions and simplifications made. |  |
| Risk of bias in individual studies | 12 | Describe methods used for assessing risk of bias of individual studies (including specification of whether this was done at the study or outcome level), and how this information is to be used in any data synthesis. | Page 5 (Quality assessment) |
| Summary measures | 13 | State the principal summary measures (e.g., risk ratio, difference in means). | Page 5-6 (Statistical analysis) |
| Synthesis of results | 14 | Describe the methods of handling data and combining results of studies, if done, including measures of consistency (e.g., I^2^) for each meta-analysis. |  |
| Risk of bias across studies | 15 | Specify any assessment of risk of bias that may affect the cumulative evidence (e.g., publication bias, selective reporting within studies). |  |
| Additional analyses | 16 | Describe methods of additional analyses (e.g., sensitivity or subgroup analyses, meta-regression), if done, indicating which were pre-specified. |  |
| RESULTS | | | |
| Study selection | 17 | Give numbers of studies screened, assessed for eligibility, and included in the review, with reasons for exclusions at each stage, ideally with a flow diagram. | Page 6 (Study selection) |
| Study characteristics | 18 | For each study, present characteristics for which data were extracted (e.g., study size, PICOS, follow-up period) and provide the citations. | Page 6-7 (Study characteristics) |
| Risk of bias within studies | 19 | Present data on risk of bias of each study and, if available, any outcome-level assessment (see Item 12). | Page 7 (Quality assessment)  Appendix 5 |
| Results of individual studies | 20 | For all outcomes considered (benefits or harms), present, for each study: (a) simple summary data for each intervention group and (b) effect estimates and confidence intervals, ideally with a forest plot. | Page 7-9 (Data synthesis and analysis), Appendix 4-6 |
| Synthesis of results | 21 | Present results of each meta-analysis done, including confidence intervals and measures of consistency. |  |
| Risk of bias across studies | 22 | Present results of any assessment of risk of bias across studies (see Item 15). |  |
| Additional analysis | 23 | Give results of additional analyses, if done (e.g., sensitivity or subgroup analyses, meta-regression [see Item 16]). | Page 9-10 (Subgroup analysis and sensitivity analysis), Appendix 7-8 |
| DISCUSSION | | | |
| Summary of evidence | 24 | Summarize the main findings including the strength of evidence for each main outcome; consider their relevance to key groups (e.g., health care providers, users, and policy makers). | Page 10 (Main findings) |
| Limitations | 25 | Discuss limitations at study and outcome level (e.g., risk of bias), and at review level (e.g., incomplete retrieval of identified research, reporting bias). | Page 10-12 (Strengths and limitations) |
| Conclusions | 26 | Provide a general interpretation of the results in the context of other evidence, and implications for future research. | Page 13 (Conclusion) |
| FUNDING | | | |
| Funding | 27 | Describe sources of funding for the systematic review and other support (e.g., supply of data); role of funders for the systematic review. | Page 1 (Funding source) |

*Note: NA, not available*

*From: Moher D, Liberati A, Tetzlaff J, Altman DG, The PRISMA Group (2009). Preferred Reporting Items for Systematic Reviews and Meta-Analyses: The PRISMA Statement. PLoS Med 6(7): e1000097. doi:10.1371/journal.pmed1000097*

# Appendix 2: Search strategies and results

Literature was searched from PubMed, Web of Science, EMBASE, Cochrane Library Database, Science Direct, SCOPUS, PsychoINFO, CINAHL, EconLit and NHS Economic Evaluation Database (NHS EED). Grey literature was searched on OpenSIGLE website and Early detection and timely INTERvention in DEMentia (INTERDEM) website (http://www.interdem.org). Search strategies were constructed by three components: “dementia”, “drug”, and “economic evaluation”. The key search terms used for each component was shown in Table A1. The detailed search strategy and results were shown in Table A2. The initial search was on April 8, 2020, and the update search was on March 24, August 14, and December 14, 2021, separately.

## Table A1. Search components and key terms

| Components | Search terms |
| --- | --- |
| Dementia | MeSH: dementia, Alzheimer Disease, cognition, Cognitive Dysfunction, Lewy Body Disease  Title: dementia, Alzheimer*, Parkinson*, cognitive impairment, cognitive decline, cognitive loss, cognitive disorder, cognitive functioning, cognition, MCI, mild cognitive impairment |
| Drug therapy | MeSH: Donepezil, Galantamine, Rivastigmine, Memantine  Title: donepezil, galantamine, rivastigmine, memantine, Exelon, drug*, medication* |
| Economic evaluation | MeSH: costs and cost analysis, economics, cost-benefit analysis, cost savings, Health Care Costs  Title: cost*, economic, cost-effectiveness, cost-utility, cost-benefit, saving*, financ*, minimization |

## Table A2. Search strategies and search results (search date: December 13, 2021)

| Database | # | Search Strategy | Hits |
| --- | --- | --- | --- |
| PubMed | 1 | (((((((((((((((dementia[MeSH Terms]) OR Alzheimer[MeSH Terms]) OR cognition[MeSH Terms]) OR Cognitive Dysfunction[MeSH Terms]) OR Lewy Body Disease[MeSH Terms]) OR dementia[Title/Abstract]) OR Alzheimer*[Title/Abstract]) OR Parkinson*[Title/Abstract]) OR cognitive impairment[Title/Abstract]) OR cognitive decline[Title/Abstract]) OR cognitive loss[Title/Abstract]) OR cognitive disorder[Title/Abstract]) OR cognitive functioning[Title/Abstract]) OR cognition[Title/Abstract]) OR mild cognitive impairment[Title/Abstract]) OR MCI[Title/Abstract] | 648,646 |
|  | 2 | ((((((((((((cost*[Title/Abstract]) OR economic*[Title/Abstract]) OR cost-effectiveness[Title/Abstract]) OR cost-utility[Title/Abstract]) OR cost-benefit[Title/Abstract]) OR cost-saving*[Title/Abstract]) OR financ*[Title/Abstract]) OR minization[Title/Abstract]) OR (Costs and Cost Analysis[MeSH Terms])) OR Economics[MeSH Terms]) OR Cost Savings[MeSH Terms]) OR Cost-Benefit Analysis[MeSH Terms]) OR Health Care Costs[MeSH Terms] | 1,431,661 |
|  | 3 | ((((Donepezil[MeSH Terms]) OR (Galantamine[MeSH Terms])) OR (Rivastigmine[MeSH Terms])) OR (Memantine[MeSH Terms]) OR (donepezil[Title] OR galantamine[Title] OR rivastigmine[Title] OR memantine[Title] OR Exelon[Title] OR drug* [Title] OR medication*[Title])) | 491,047 |
|  | 4 | #1 AND #2 AND #3 | 1,076 |
| EMBASE | 1 | (dementia or Alzheimer* or Parkinson or cognitive or cognition or MCI or mild cognitive impairment).mp. [mp=title, abstract, heading word, drug trade name, original title, device manufacturer, drug manufacturer, device trade name, keyword, floating subheading word, candidate term word] | 1,158,647 |
|  | 2 | (cost* or economic or cost-effectiveness or cost-utility or cost-benefit or saving* or financ* or minimization).m_titl. | 254,593 |
|  | 3 | (donepezil or galantamine or rivastigmine or memantine or Exelon or drug* or medication*).mp. [mp=title, abstract, heading word, drug trade name, original title, device manufacturer, drug manufacturer, device trade name, keyword, floating subheading word, candidate term word] | 12,103,535 |
|  | 4 | #1 AND #2 AND #3 | 1,760 |
| Science Direct | 1 | Title: (dementia OR Alzheimer* OR cognitive OR cognition OR MCI OR "cognitive impairment") AND (cost* OR economic OR cost-effectiveness OR cost-utility OR cost-benefit OR saving* OR financ* OR minimization | 921 |
| SCOPUS | 1 | TITLE-ABS-KEY ( dementia OR alzheimer* OR parkinson* OR "cognitive impairment" OR "cognitive decline" OR "cognitive loss" OR "cognitive disorder" OR "cognitive functioning" OR cognition OR mci OR "mild cognitive impairment" ) AND TITLE (cost* OR economic OR cost-effectiveness OR cost-utility OR cost-benefit OR saving* OR financ* OR minimization ) AND TITLE (donepezil or galantamine or rivastigmine or memantine or Exelon or drug* or medication* ) | 203 |
| Web of Science | 1 | TS= (dementia OR Alzheimer* OR Parkinson* OR "cognitive impairment" OR "cognitive decline" OR "cognitive loss" OR "cognitive disorder" OR "cognitive functioning" OR cognition OR MCI OR "mild cognitive impairment") | 749,249 |
|  | 2 | TI= (cost* OR economic OR cost-effectiveness OR cost-utility OR cost-benefit OR saving* OR financ* OR minimization) | 804,131 |
|  | 3 | TS= (donepezil OR galantamine OR rivastigmine OR memantine OR Exelon OR drug* OR medication*) | 2,293,028 |
|  | 4 | #1 AND #2 AND #3 | 588 |
| Cochrane Library Database | 1 | (dementia OR Alzheimer* OR Parkinson* OR "cognitive impairment" OR "cognitive decline" OR "cognitive loss" OR "cognitive disorder" OR "cognitive functioning" OR cognition OR MCI OR "mild cognitive impairment" in Title Abstract Keyword) AND (cost* OR economic OR cost-effectiveness OR cost-utility OR cost-benefit OR saving* OR financ* OR minimization in Record Title) AND (donepezil OR galantamine OR rivastigmine OR memantine OR Exelon OR drug* OR medication* in Title Abstract Keyword) | 397 |
| PsycINFO | 1 | (dementia or Alzheimer* or Parkinson or cognitive or cognition or MCI or mild cognitive impairment).mp. [mp=title, abstract, heading word, table of contents, key concepts, original title, tests & measures, mesh] | 701,220 |
|  | 2 | (cost* OR economic OR cost-effectiveness OR cost-utility OR cost-benefit OR saving* OR financ* OR minimization).m_titl. | 37,301 |
|  | 3 | (donepezil OR galantamine OR rivastigmine OR memantine OR Exelon OR drug* OR medication*).m_titl. | 66,441 |
|  | 4 | #1 and #2 and #3 | 77 |
| CINAHL | 1 | TI ( dementia OR Alzheimer* OR Parkinson* OR "cognitive impairment" OR "cognitive decline" OR "cognitive loss" OR "cognitive disorder" OR "cognitive functioning" OR cognition OR MCI OR "mild cognitive impairment" ) AND TI ( cost* OR economic OR cost-effectiveness OR cost-utility OR cost-benefit OR saving* OR financ* OR minimization ) AND TI ( donepezil OR galantamine OR rivastigmine OR memantine OR Exelon OR drug* OR medication*) | 66 |
| EconLit | 1 | ( dementia OR Alzheimer* OR Parkinson* OR "cognitive impairment" OR "cognitive decline" OR "cognitive loss" OR "cognitive disorder" OR "cognitive functioning" OR cognition OR MCI OR "mild cognitive impairment" ) AND ( cost* OR economic OR cost-effectiveness OR cost-utility OR cost-benefit OR saving* OR financ* OR minimization ) AND ( donepezil OR galantamine OR rivastigmine OR memantine OR Exelon OR drug* OR medication* ) | 61 |
| CRD (NHS EED, DARE, HTA) | 1 | Any field:( dementia OR Alzheimer* OR Parkinson* OR "cognitive impairment" OR "cognitive decline" OR "cognitive loss" OR "cognitive disorder" OR "cognitive functioning" OR cognition OR MCI OR "mild cognitive impairment" ) AND title:( cost* OR economic OR cost-effectiveness OR cost-utility OR cost-benefit OR saving* OR financ* OR minimization ) | 233 |
| OpenSIGLE (http://www.opengrey.eu/) | 1 | dementia cost* | 10 |
|  | 2 | dementia cost-effectiveness | 0 |
|  | 3 | dementia economic | 4 |
|  | 4 | alzheimer* cost* | 13 |
|  | 5 | alzheimer* cost-effectiveness | 1 |
|  | 6 | alzheimer* economic | 6 |
| INTERDEM | 1 | Path: Publications – Method articles – Cost effectiveness studies | 27 |
| Chinese Biomedical Literature Database | 1 | ("痴呆"[中文标题:智能] OR "海默"[中文标题:智能] OR "认知功能"[中文标题:智能] OR "认知障碍"[中文标题:智能] OR "脑退化"[中文标题:智能] OR "dementia"[英文标题:智能] OR "cognition"[英文标题:智能] OR "cognitive"[英文标题:智能] OR "MCI"[英文标题:智能]) AND ("经济"[中文标题:智能] OR "成本"[中文标题:智能] OR "负担"[中文标题:智能] OR "费用"[中文标题:智能] OR "cost*"[英文标题:智能] OR "economic"[英文标题:智能] OR "saving*"[英文标题:智能] OR "financ*"[英文标题:智能] OR "minimization"[英文标题:智能] OR "cost-effectiveness"[英文标题:智能] OR "cost-benefit"[英文标题:智能] OR "cost-utility"[英文标题:智能]) | 260 |
| CNKI | 1 | (TI='痴呆' OR TI='海默' OR TI='认知功能' OR TI='认知障碍' OR TI='脑退化') AND (TI='经济' OR TI='成本' OR TI='负担' OR TI='费用')  Language: limited to Chinese | 258 |
| Wang Fang Database | 1 | 题名:("痴呆" or "海默" or "认知功能" or "认知障碍" or "脑退化" or "dementia" or "Alzheimer" or "cognition" or "cognitive" or "MCI") and 主题:("经济" or "成本" or "负担" or "费用" or "cost" or "economic" or "savingand" or "financeand" or "minimization" or "cost-effectiveness" or "cost-benefit" or "cost-utility")  Language: limited to Chinese | 5,531 |

# Appendix 3: PICOS criteria and study selection

## Table A3. PICOS criteria for inclusion and exclusion of studies

| PICOS items | Contents |
| --- | --- |
| Participants | People with any type of dementia (Alzheimer’s disease, vascular dementia, Parkinson’s disease dementia, frontotemporal dementia, or Lewy body’s dementia) at any stage (mild, moderate, or severe) are eligible. People with unintended causes of cognitive impairment that are out of scope of this study are excluded (e.g., dementia caused by brain injury, infection, HIV, substance abuse and improper medication use). |
| Interventions | Pharmaceutical interventions: consumption or application of a substance, including drugs (e.g., cholinesterase inhibitors, memantine, tacrine), vitamins and food supplements, herbal medicines and homeopathic remedies, oxygen, and acupuncture |
| Comparisons | Usual care, treatment as usual, placebo, no treatment, and any alternative interventions |
| Outcomes | Monetary cost: cost of medications, direct medical cost, direct nonmedical cost, and indirect cost (productivity loss, informal care)  Health outcomes in five domains: (1) cognitive functioning, (2) activity of daily living, (3) behavioural and psychiatric symptoms of dementia (BPSDs), (4) global deterioration of disease, (5) health-related quality of life (HRQoL).  Economic evidence: cost-effectiveness ratio (C/E), incremental cost-effectiveness ratio (ICER) |
| Study Design | Design: randomised controlled trials (RCTs), non-randomised controlled trials, quasi-experimental designs, and observational studies. Modelling-based studies, case-control studies, case series and case reports are excluded.  Type of economic evaluation: cost-effectiveness analysis (CEA), cost-utility analysis (CUA), cost-benefit analysis (CBA), cost-minimization analysis (CMA), cost-consequence analysis (CCA). Modelling studies are not considered. Perspectives of analysis were not restricted.  Settings: not limited |

## Figure A1. PRISMA flow diagram

Studies included in systematic review

(n = 10)

Full-text articles excluded

(n =134)

Duplicate: 23

Review, summary and editorial: 18

Protocol: 6

Conference: 11

Model-based: 52

Insufficient data or measurements: 11

Others: 13

Full-text articles assessed for eligibility

(n = 144)

Additional records identified through other sources

(n = 93)

Records identified from database

(n = 11492)

(PUBMED: 1076; EMBASE: 1760; SCIENCE DIRECT:921; SCOPUS: 203; WEB OF SCIENCE: 588; Cochrane Library Database: 397; PsycINFO: 77; CINAHL: 66; EconLit: 61; CDR: 233; OpenSIGLE: 34; INTERDEM: 27; CBM: 260; CNKI: 258; WANGFANG: 5531)

Screening

Eligibility

Records combined

(n = 11585)

Records after duplicates removed

(n = 10616)

Records excluded

(n = 10472)

Irrelevance: 10286

Review: 40

Protocol: 21

Not drug therapy: 55

Conference, abstract or no full text:51

No access: 19

Identification

Included

# Appendix 4: Characteristics of eligible studies

## Table A4. Basic characteristics of 10 included studies

|  | | Study, country | Study design | Participants, setting and funding | Time horizon, Intervention (IG), Comparator (CG) | Perspective and cost data ^a^ | Measure of health outcome | Additional reference |
| --- | --- | --- | --- | --- | --- | --- | --- | --- |
| *Drugs aiming at symptoms of cognitive decline and global deterioration* | | | | | | | |  |
|  | | Bachynsky (2000), Canada (29) | RCT  CEA | Number: 550  Type: AD or VaD  Severity: MIL to MOD  Mean age: 71.5  Setting: community  Funding: Hoechst Marion Roussel Canada Research, Inc. | Time horizon: 48 weeks  IG: Propentofylline (300 mg at a time, three times per day)  CG: placebo | Perspective: (1) public sector (Ministry of Health), (2) societal  Component: (1) health care utilization of patients, (2) informal care, (3) cost of studied drugs  Measurement: (1) Resource Utilization Groups, (2) Caregiver’s Activity Time Survey | Cognition: MMSE, SKT  ADLs: NAB  Global deterioration: GDS, GBS, CGI | (41-43) |
|  | | Courtney (2004), UK (23) | RCT  CEA | Number: 565  Type: AD with or without VaD  Severity: MIL to MOD  Mean age: 75.5  Setting: memory clinic  Funding: NHS ExecutiveR&D (West Midlands) | Time horizon: 60 weeks  IG: Donepezil (5 or 10 mg/day)  CG: placebo | Perspective: health and social care  Component: (1) health care utilization of patients  Measurement: carer questionnaire | Cognition: MMSE, ADAS-cog  ADLs: BADL  BPSD: NPI  Destination: risk of institutionalisation |  |
|  | | Feldman (2004), Canada, Australia, France (24) | RCT  CCA | Number: 290  Type: AD  Severity: MOD to SEV  Mean age: 73.5  Setting: community, retirement home, nursing home, extended care  Funding: supported by Pfizer Inc., and Eisai Co. Ltd. | Time horizon: 24 weeks  IG: Donepezil (5 or 10 mg/day)  CG: placebo | Perspective: societal (dyads)  Cost Component: (1) AD-related health care utilization of patients, (2) informal care, (3) health care utilization of informal caregivers, (4) cost of studied drugs  Measurement: (1) CAUST, (2) carer interview | Cognition: MMSE, SIB  ADLs: Lawton’s IADL, PSMS+, DAD  Global deterioration: CIBIC+, FRS  BPSD: NPI | (44-46) |
|  | | Knapp (2017), UK (30) | RCT  CEA  CUA | Number: 295  Type: AD receiving donepezil for over 3 months  Severity: MOD to SEV  Mean age: 77.1  Setting: community  Funding: UK Medical Research Council, UK Alzheimer's Society, National Institute for Health Research, the NIHR School for Social Care Research | Time horizon: 52 weeks  IG1: Donepezil (10 mg/day) + Memantine (20 mg/day)  IG2: Donepezil (10 mg/day)  IG3: Memantine (20 mg/day)  CG: placebo | Perspective: (1) health and social care, (2) societal  Component: (1) health care utilization of patients, (2) informal care, (3) cost of studied drugs  Measurement: CSRI | Cognition: MMSE  ADLs: BADLS  BPSD: NPI  Health utility and QALY: EQ-5D(P), DEMQOL(P) | (47) |
|  | | Suh (2008), South Korea (26) | Quasi-experimental design (RCT extensive with community cohorts)  CCA | Number: 138  Type: AD  Severity: MIL to MOD  Mean age: 75.3  Setting: community  Funding: Janssen Korea Pharmaceutical | Time horizon: 52 weeks  IG: Galantamine (orally 8, 16, or 24 mg/day)  CG: no treatment | Perspective: societal  Component: (1) health care utilization of patients, (2) informal care and indirect cost of caregivers, (3) cost of studied drugs  Measurement: (1) CSRI, (2) RUD | Cognition: ADAS-cog  ADLs: DAD  Global deterioration: GDS  BPSD: BEHAVE-AD | (48) |
|  | | Willan (2006), twelve countries across Europe and Canada (27) | RCT  CEA  CUA | Number: 541  Type: Parkinson’s disease dementia for at least 2 years  Severity: MIL to MOD  Mean age: 73  Setting: community  Funding: Novartis | Time horizon: 24 weeks  IG: Rivastigmine (6 or 12 mg/day)  CG: placebo | Perspective: societal  Component: (1) health care utilization of patients, (2) informal care, (3) cost of studied drugs  Measurement: study case report forms | Cognition: MMSE, ADAS-cog, CDR power of attention tests, D-KEFS-VFT, CDT  ADLs: ADCS-ADL  Global deterioration: ADCS-CGIC  BPSD: NPI-10  Health utility and QALY: MMSE-based | (49) |
|  | | Wimo (2003a), Denmark, Finland, Norway, Sweden, and The Netherlands (30) | RCT  CCA | Number: 286  Type: AD  Severity: MIL to MOD  Mean age: 72.5  Setting: community  Funding: Pfizer Inc | Time horizon: 52 weeks  IG: Donepezil (5 mg/day for 28 days followed by 10 mg/day)  CG: placebo | Perspective: societal (dyads)  Component: (1) health care utilization of patients, (2) informal care, (3) health care utilization of informal caregivers, (4) cost of studied drugs  Measurement: RUD | Cognition: MMSE  ADLs: PDS  Global deterioration: GBS, GDS | (47) |
|  | | Wimo (2003b), US (28) | RCT  CCA | Number: 252  Type: AD  Severity: MOD to SEV  Mean age: 59% older than 75 years  Setting: community or institution  Funding: not stated | Time horizon: 28 weeks  IG: Memantine (5 or 10mg at a time, two times per day)  CG: placebo | Perspective: societal (dyads)  Component: (1) health care utilization of patients and caregivers, (2) informal care and indirect cost of caregivers  Measurement: RUD | Cognition: MMSE, SIB  ADLs: ADL, FAST  Global deterioration: CIBIC+  BPSD: NPI | (50) |
| *Drugs aiming at psychological and behavioural symptoms of people with dementia* | | | | | | | |  |
|  |  | | RCT  CEA  CUA | Number: 326  Type: AD with depression  Severity: not specified  Mean age: 79  Setting: community (psychiatry service, research clinic, tertiary care)  Funding: National Institute for Health Research, pharmaceutical companies | Time horizon: 39 weeks  IG1: Mirtazapine (45mg/day)  IG2: Sertraline (150mg/day)  CG: placebo | Perspective: (1) health and social care, (2) societal  Component: (1) healthcare utilization of patients, (2) informal care, (3) cost of studied drugs  Measurement: CSRI | Cognition: MMSE  ADLs: BADL  BPSD: NPI, CSDD  Health utility and QALY: EQ-5D(S+P), DEMQOL(S+P) | (33) |
|  | | Rosenheck (2007), US (31) | RCT  CEA  CUA  CBA | Number: 421  Type: AD with severe delusions, hallucinations, aggression, or agitation  Severity: not specified  Mean age: 77.9  Setting: at home or assisted living  Funding: National Institute of Mental Health, AstraZeneca, Forest Pharmaceuticals, Janssen Pharmaceutica, Eli Lilly | Time horizon: 36 weeks  IG1: Olanzapine (2.5 or 5.0mg/day)  IG2: Risperidone (0.5 or 1.0mg/day)  IG3: Quetiapine (25 or 50mg/day)  CG: placebo | Perspective: health and social care  Component: (1) healthcare utilization of patients, (2) cost of studied drugs  Measurement: carer questionnaire | ADLs: ADCS-ADL, AD Dependence Scale  Global deterioration: CGIC  Health utility and QALY: HUI-3(S), ADRQOL(S) | (51) |
| *Study included in sensitivity analysis (Galantamine)* | | | | | | | | |
|  | | Wimo (2012), Sweden (34) | RCT + Population-based survey  Cost analysis | Number: 80  Type: AD  Severity: mild to moderate  Mean age: 72  Setting: outpatient clinic  Funding: Janssen Research  Foundation, Beerse, Belgium | Time horizon: 28 weeks  IG: Galantamine (24 or 32 mg/day)  CG: placebo | Perspective: (1) health and social care, (2) societal  Component: (1) healthcare utilization of patients, (2) informal care, (3) cost of studied drugs  Measurement: regression analysis linked to ADL (KNP’s resource utilization and cost database) | ADLs: DAD, Katz’ Index of Independence in ADL | (52) |

a. Dyads: cost collection considered healthcare utilization of both patients and caregivers.

*Notes: AD, Alzheimer's Disease; ADAS-cog, the Alzheimer's Disease Assessment Scale-Cognitive Subscale; ADCS-ADL, Alzheimer’s Disease Cooperative Study Activities of Daily Living Scale; ADCS-CGIC, Alzheimer’s Disease Cooperative Study - Clinician’s Global Impression of Change; ADL, activity of daily living; ADRQOL, Alzheimer’s Disease Related Quality of Life Scale; BADL, Bristol activities of daily living scale; BEHAVE-AD, Behavior Pathology in Alzheimer’s disease Rating Scale; BPSD, behavioural and psychological symptoms of dementia; CAUST, Canadian Utilization of Services Tracking questionnaire; CBA, cost-benefit analysis; CCA, cost-consequence analysis; CDR, Cognitive Drug Research (computerized assessment system power of attention tests); CDT, clock drawing test; CEA, cost-effectiveness analysis; CGI, Clinical Global Impressions; CGIC, Clinical Global Impression of Change; CIBIC+, Clinician’s Interview-Based Impression of Change with caregiver input – plus; CSDD, Cornell Scale for Depression in Dementia; CSRI, Client Service Receipt Inventory; CUA, cost-utility analysis; DAD, Disability Assessment for Dementia; DEMQOL,* *Dementia Quality of Life; D-KEFS, Delis–Kaplan Executive Function System; EQ-5D,* *European Quality of Life-5 Dimensions; FAST, Functional Assessment Staging; FRS, Functional Rating Scale; GBS, Gottfries-Brine-Steen scale; GDS, Global Deterioration Scale;* *HUI, Health Utility Index; IADL: Instrumental Activities of Daily Living; MIL, mild; MMSE, Mini–Mental State Examination; MOD, moderate; NAB, Nürnberger Altersbeobachtungsskala (NAB questionnaire); NPI,* *Neuropsychiatric Inventory Questionnaire; PDS, Progressive Deterioration Scale; PSMS+, modified Physical Self-Maintenance Scale; P, proxy-rated; QALY, quality-adjusted life year; RCT, randomized controlled trial; RMBPC: Revised Memory and Behaviour Problems Checklist; RUD, Resource Utilization in Dementia; S, self-rated; SIB, Severe impairment battery; SKT, Syndrome Short Test;* *VaD, vascular dementia; VFT, Verbal Fluency Test*

## Table A5. Summary of study characteristics

|  | Number (n) | Percentage (%) |
| --- | --- | --- |
| Total number of studies | 10 | 100 |
| ***Characteristics of study*** |  |  |
| Year of publication |  |  |
| before 2005 | 5 | 50.0 |
| 2005-2009 | 3 | 30.0 |
| 2010-2014 | 1 | 10.0 |
| 2015-2021.8 | 1 | 10.0 |
| Location |  |  |
| UK | 3 | 30.0 |
| North America (US, Canada) | 3 | 30.0 |
| South Korea | 1 | 10.0 |
| Multi-centres ^a^ | 3 | 30.0 |
| Setting of study |  |  |
| Community ^b^ | 7 | 70.0 |
| Community + Institution ^c^ | 3 | 30.0 |
| Study design |  |  |
| Randomized Control Trial | 9 | 90.0 |
| Quasi-experimental design | 1 | 10.0 |
| Time horizon of study (intervention) |  |  |
| 4-6 months | 2 (2) | 20.0 (20.0) |
| 7-12 months | 7 (8) | 70.0 (80.0) |
| >12 months | 1 (0) | 10.0 (0.0) |
| Source of funding |  |  |
| Industrial entities | 4 | 40.0 |
| Non-industrial entities | 2 | 20.0 |
| Both non-industrial and industrial entities | 3 | 30.0 |
| Not stated | 1 | 10.0 |
| ***Characteristics of participants*** |  |  |
| Total number of participants | 3664 | |
| Mean/Median | 366/311 | |
| <200 | 1 | 10.0 |
| 200-299 | 4 | 40.0 |
| 300-499 | 2 | 20.0 |
| >=500 | 3 | 30.0 |
| Distribution of mean or median age |  |  |
| 70-74 | 4 | 40.0 |
| 75-79 | 6 | 60.0 |
| Type of dementia |  |  |
| Alzheimer’s disease (AD) | 7 | 70.0 |
| Parkinson’s disease dementia | 1 | 10.0 |
| AD or Vascular dementia | 2 | 20.0 |
| Severity of dementia |  |  |
| Mild to moderate severity | 5 | 50.0 |
| Moderate to severe severity | 3 | 30.0 |
| Not limited or not specified | 2 | 20.0 |
| ***Characteristics of intervention*** |  |  |
| Type of intervention |  |  |
| Cholinesterase inhibitors or memantine | 7 | 70.0 |
| Psychotropic medicine | 2 | 20.0 |
| Others (Propentofylline) | 1 | 10.0 |
| Type of controls |  |  |
| Placebo or No treatment | 7 | 70.0 |
| Alternative drugs + Placebo | 3 | 30.0 |
| ***Characteristics of outcome*** |  |  |
| Type of economic evaluation |  |  |
| Cost-Effectiveness Analysis (CEA) | 2 | 20.0 |
| Cost-Utility Analysis (CUA) | 0 | 0.0 |
| CUA and CEA | 4 | 40.0 |
| Cost-Benefit Analysis | 0 | 0.0 |
| Others (Cost-consequence analysis) | 4 | 40.0 |
| Measurement of outcome |  |  |
| Cognitive functions | 9 | 90.0 |
| Activity of living | 10 | 100.0 |
| Global deterioration | 7 | 70.0 |
| Behavioural and psychological symptoms | 7 | 70.0 |
| Institutionalization | 1 | 10.0 |
| Quality of life (reporting QALY) | 4 (4) | 40.0 (40.0) |
| Perspective |  |  |
| Societal perspective only ^d^ | 5 | 50.0 |
| Health care service or healthcare payer only | 2 | 20.0 |
| Both societal and health care perspective | 3 | 30.0 |
| Including health utilization of caregivers |  |  |
| Yes | 3 | 30.0 |
| No | 7 | 70.0 |

a. Multi-centres included European countries, Australia, and Canada.

b. Community settings include general practitioner, memory clinic, day care centre, neurology clinic and tertiary care.

c. Institutional settings include assisted living facilities, retirement home, residential home, nursing home, dementia care home and hospital ward.

d. Societal perspectives include cost external to formal service (productivity losses, informal caregiving).

# Appendix 5: Quality assessment

## Table A6. Quality assessment of included studies

(1) Randomized Control Trial (using RoB2)

| Study | Randomization process | Deviations from intended interventions | Missing outcome data | Measurement of outcome | Selection of the reported result | Overall bias |
| --- | --- | --- | --- | --- | --- | --- |
| Bachynsky 2000 | + | + | + | + | + | + |
| Banerjee 2013 | + | + | - | + | + | - |
| Courtney 2004 | + | + | + | + | + | + |
| Feldman 2004 | + | + | ? | ? | + | ? |
| Knapp 2017 | + | + | + | + | + | + |
| Rosenheck 2007 | + | + | + | + | - | + |
| Willan 2006 | + | + | + | + | + | + |
| Wimo 2003a | + | + | ? | + | + | ? |
| Wimo 2003b | ? | ? | + | + | ? | ? |
| Wimo 2012 (Only for sensitivity analysis) | + | + | + | + | + | + |

Legends: +, low risk of bias; -, high risk of bias; ?, some concerns

(2) Non-Randomized Study (using ROBINS-I)

| Study | Confounding | Selection of participants | Classification of interventions | Deviations from intended interventions | Missing data | Measurement of outcomes | Selection of the reported result | Overall bias |
| --- | --- | --- | --- | --- | --- | --- | --- | --- |
| Suh 2008 | 0 | - | + | 0 | - | - | + | - |

Legends: +, low risk of bias; 0, moderate risk of bias; -, serious risk of bias

*Notes: RoB2, the version 2 of Cochrane risk-of-bias tool; ROBINS-I, the Risk of Bias in Non-randomized studies of Interventions tool*

## Figure A2. Summary of risk of bias of included studies (RCTs, n=9)

## Table A7. Methodological quality of economic evaluation using CHEC list

| Study | Q1 | Q2 | Q3 | Q4 | Q5 | Q6 | Q7 | Q8 | Q9 | Q10 | Q11 | Q12 | Q13 | Q14 | Q15 | Q16 | Q17 | Q18 | Q19 | Yes% |
| --- | --- | --- | --- | --- | --- | --- | --- | --- | --- | --- | --- | --- | --- | --- | --- | --- | --- | --- | --- | --- |
| Bachynsky 2000 | Y | Y | Y | Y | Y | Y | Y | Y | Y | Y | Y | Y | Y | na | Y | Y | Y | N | N | 89% |
| Banerjee 2013 | Y | Y | Y | Y | Y | Y | Y | Y | Y | Y | Y | Y | Y | na | Y | Y | Y | N | Y | 95% |
| Courtney 2004 | Y | Y | Y | Y | Y | Y | N | Y | Y | Y | Y | Y | N | N | Y | Y | Y | Y | Y | 84% |
| Feldman 2004 | Y | N | Y | Y | Y | Y | N | Y | Y | N | N | N | N | na | Y | Y | Y | N | Y | 63% |
| Knapp 2017 | Y | Y | Y | Y | Y | Y | Y | Y | Y | Y | Y | Y | Y | na | Y | Y | Y | Y | Y | 100% |
| Rosenheck 2007 | Y | N | Y | Y | Y | Y | Y | Y | Y | Y | Y | Y | Y | na | Y | Y | Y | N | Y | 89% |
| Suh 2008 | Y | Y | Y | Y | Y | Y | Y | Y | Y | Y | Y | Y | N | na | Y | Y | Y | N | Y | 89% |
| Willan 2006 | Y | Y | Y | Y | Y | Y | Y | Y | Y | Y | Y | Y | Y | na | Y | Y | Y | Y | Y | 95% |
| Wimo 2003a | Y | Y | Y | Y | Y | Y | Y | Y | Y | Y | Y | Y | N | na | Y | Y | Y | N | Y | 89% |
| Wimo 2003b | Y | Y | Y | Y | Y | Y | Y | Y | Y | Y | Y | Y | N | na | Y | Y | Y | N | Y | 89% |
| Wimo 2012 (Only for sensitivity analysis) | Not applicable because cost collection was based on regression analysis | | | | | | | | | | | | | | | | | | | |

Signalling questions of CHEC list (17):

Q1. Is the study population clearly described?

Q2. Are competing alternatives clearly described?

Q3. Is a well-defined research question posed in answerable form?

Q4. Is the economic study design appropriate to the stated objective?

Q5. Is the chosen time horizon appropriate in order to include relevant costs and consequences?

Q6. Is the actual perspective chosen appropriate?

Q7. Are all important and relevant costs for each alternative identified?

Q8. Are all costs measured appropriately in physical units?

Q9. Are costs valued appropriately?

Q10. Are all important and relevant outcomes for each alternative identified?

Q11. Are all outcomes measured appropriately?

Q12. Are outcomes valued appropriately?

Q13. Is an incremental analysis of costs and outcomes of alternatives performed?

Q14. Are all future costs and outcomes discounted appropriately?

Q15. Are all important variables, whose values are uncertain, appropriately subjected to sensitivity analysis?

Q16. Do the conclusions follow from the data reported?

Q17. Does the study discuss the generalizability of the results to other settings and patient/client groups?

Q18. Does the article indicate that there is no potential conflict of interest of study researcher(s) and funder(s)?

Q19. Are ethical and distributional issues discussed appropriately?

Response to questions: Y, yes indicating high quality; N, no indicating low quality; n.a, not applicable; Yes%, % of items meeting high quality among all items

## Figure A3. Summary of quality of economic evaluation in included studies

# Appendix 6: Costs and effects of interventions

## Table A8. Effects of intervention on health outcomes extracted from included studies ^a^

| Study | Intervention | Comparison | Cognition | Activity function | Global disease progression | BPSD | Health utility score | QALY |
| --- | --- | --- | --- | --- | --- | --- | --- | --- |
| *Drugs aiming at symptoms of cognitive decline and global deterioration* | | | | | | | | |
| Bachynsky (2000) | Propentofylline | Placebo | MMSE: 0.228 (0.087)  SKT: 0.228 (0.087) | NAB: 0.212 (0.087) | GDS: 0.287 (0.087)  GBS: 0.144 (0.087)  CGI: 0.189 (0.087) |  |  |  |
| Courtney (2004) | Donepezil | Placebo | MMSE: 0.338 (0.085) | BADL: 0.307 (0.085) |  | NPI: 0.044 (0.084) |  |  |
| Feldman (2004) | Donepezil | Placebo | MMSE: 0.481 (0.124)  SIB: 0.481 (0.124) | DAD: 0.477 (0.123)  Lawton’s IADL: 0.477 (0.123)  PSMS+: 0.388 (0.122) | CIBIC+: 0.467 (0.120)  FRS: 0.446 (0.120) | NPI: 0.420 (0.120) |  |  |
| Knapp (2017) | Donepezil | Placebo | MMSE: 1.468 (0.262) | BADL: 1.112 (0.250) |  | NPI: 0.416 (0.235) | DEMQOL-proxy: -0.323 (0.234) | QALY (EQ-5D): 0.637 (0.238) |
|  | Memantine | Placebo | MMSE: 0.794 (0.207) | BADL: 0.477 (0.203) |  | NPI: 0.618 (0.205) | DEMQOL-proxy: 0.224 (0.201) | QALY (EQ-5D): 0.327 (0.202) |
|  | Donepezil + Memantine | Donepezil alone | MMSE: 0.376 (0.203) | BADL: 0.118 (0.202) |  | NPI: 0.568 (0.205) | DEMQOL-proxy: 0.249 (0.203) | QALY (EQ-5D): 0.092 (0.202) |
| Suh (2008) | Galantamine | No treatment | ADAS-cog: 0.683 (0.175) | DAD: 0.683 (0.175) |  |  |  |  |
| Willan (2006) | Rivastigmine | Placebo | MMSE: 0.209 (0.096)  ADAS-cog: 0.318 (0.097)  CDR power of attention tests: 0.252 (0.097)  D-KEFS VFT: 0.318 (0.097)  CDT: 0.224 (0.096) | ADCS-ADL: 0.222 (0.095) | ADCS-CGIC: 0.258 (0.096) | NPI: 0.222 (0.095) |  | QALY (MMSE): 0.141 (0.093) |
| Wimo (2003a) | Donepezil | Placebo | MMSE: 0.403 (0.122) | PDS: 0.286 (0.146) | GBS: 0.2314 (0.120) |  |  |  |
| Wimo (2003b) | Memantine | Placebo | SIB: 0.470 (0.152) | ADL inventory: 0.448 (0.151)  FAST: 0.407 (0.151) | CIBIC+: 0.337 (0.150) |  |  |  |
| *Drugs aiming at psychological and behavioural symptoms of people with dementia* | | | | | | | | |
| Banerjee (2013) | Mirtazapine | Sertraline | MMSE: -0.212 (0.237) | BADL: 0.075 (0.236) |  | NPI: 0.329 (0.238)  CSDD: 0.221 (0.237) | EQ-5D: 0.206 (0.275)  DEMQOL: 0.231 (0.276)  EQ-5D-proxy: -0.068 (0.275)  DEMQOL-proxy: 0.117 (0.275) | QALY (EQ-5D): 0.221 (0.276) |
|  | Mirtazapine | Placebo | MMSE: -0.575 (0.230) | BADL: -0.206 (0.226) |  | NPI: 0.141 (0.225)  CSDD: 0.191 (0.226) | EQ-5D: -0.077 (0.270)  DEMQOL: -0.004 (0.227)  EQ-5D-proxy: -0.093 (0.270)  DEMQOL-proxy: 0.437 (0.273) | QALY (EQ-5D): 0.492 (0.274) |
|  | Sertraline | Placebo | MMSE: -0.188 (0.232) | BADL: -0.280 (0.233) |  | NPI: -0.180 (0.226)  CSDD: -0.013 (0.225) | EQ-5D: -0.277 (0.269)  DEMQOL: -0.231 (0.268)  EQ-5D-proxy: -0.022 (0.267)  DEMQOL-proxy: 0.316 (0.269) | QALY (EQ-5D): 0.268 (0.269) |
| Rosenheck (2007) | Olanzapine | Placebo |  | ADCS-ADL: -0.411 (0.132)  AD Dependence Scale: -0.211 (0.177) | CGIC minimal improvement: 0.258 (0.177) |  | ADRQOL: 0.035 (0.177) | QALY (HUI-3): -0.125 (0.177) |
|  | Risperidone | Placebo |  | ADCS-ADL: -0.113 (0.137)  AD Dependence Scale: -0.127 (0.182) | CGIC minimal improvement: 0.188 (0.182) |  | ADRQOL: -0.114 (0.182) | QALY (HUI-3): 0.129 (0.182) |
|  | Quetiapine | Placebo |  | ADCS-ADL: -0.065 (0.133)  AD Dependence Scale: -0.213 (0.179) | CGIC minimal improvement: 0.120 (0.179) |  | ADRQOL: 0.008 (0.179) | QALY (HUI-3): 0.063 (0.179) |

a. Effects of intervention were reported based on SMD(SE) (Cohen’s d effect) and different domains of measure. Positive value in effect size indicates improvement in the health outcome.

*Notes: ADAS-cog, the Alzheimer's Disease Assessment Scale-Cognitive Subscale; ADCS-ADL, Alzheimer’s Disease Cooperative Study Activities of Daily Living Scale; ADCS-CGIC, Alzheimer’s Disease Cooperative Study - Clinician’s Global Impression of Change; ADL, activity of daily living; ADRQOL, Alzheimer’s Disease Related Quality of Life Scale; BADL, Bristol activities of daily living scale; BEHAVE-AD, Behavior Pathology in Alzheimer’s disease Rating Scale; BPSD, behavioural and psychological symptoms of dementia; CDR, Cognitive Drug Research (computerized assessment system power of attention tests); CDT, clock drawing test; CGI, Clinical Global Impressions; CGIC, Clinical Global Impression of Change; CIBIC+, Clinician’s Interview-Based Impression of Change with caregiver input – plus; CSDD, Cornell Scale for Depression in Dementia; DAD, Disability Assessment for Dementia; DEMQOL, Dementia Quality of Life; D-KEFS, Delis–Kaplan Executive Function System; EQ-5D, European Quality of Life-5 Dimensions; FAST, Functional Assessment Staging; FRS, Functional Rating Scale; GBS, Gottfries-Brine-Steen scale; GDS, Global Deterioration Scale; HUI, Health Utility Index; IADL: Instrumental Activities of Daily Living; MMSE, Mini–Mental State Examination; NAB, Nürnberger Altersbeobachtungsskala (NAB questionnaire); NPI, Neuropsychiatric Inventory Questionnaire; PDS, Progressive Deterioration Scale; PSMS+, modified Physical Self-Maintenance Scale; QALY, quality-adjusted life year; SIB, Severe impairment battery; SKT, Syndrome Short Test; VFT, Verbal Fluency Test*

## Table A9. Cost information extracted from included studies ^a^

| Study | Intervention | Comparison | Follow-up (weeks) | Currency (Price Year) | Incremental cost extracted from studies  (before standardization) ^b^ | | | | Conversion Factor ^c^ | Standardized incremental cost  (USD 2020) | |
| --- | --- | --- | --- | --- | --- | --- | --- | --- | --- | --- | --- |
|  |  |  |  |  | Cost of medications | Healthcare service | Informal/Indirect cost | Total cost |  | Healthcare service perspective (SE) | Societal perspective (SE) |
| *Drugs aiming at symptoms of cognitive decline and global deterioration* | | | | | | | | | | | |
| Bachynsky (2000) | Propentofylline | Placebo | 48w | CAN (1994) | 1618 | -22 ^d^ | -1142 | 454 | 1.36 | 2171 (554) | 617 |
| Courtney (2004) | Donepezil | Placebo | 39w | GBP (2000) | N.R | 498 | N.R | 498 | 2.12 | 1056 (746) | N.A |
| Feldman (2004) | Donepezil | Placebo | 24w | CAN (1998) | 842 | -536 ^e^ | -233 | 74 | 1.29 | N.A | 96 (1120) |
| Knapp (2017) | Donepezil | Placebo | 52w | GBP (2013) | 21 | -3022 ^d^ | 2276 | -2669 ^f^ | 1.62 | -630 (2616) | -4324 (3742) |
|  | Memantine | Placebo | 52w | GBP (2013) | 26 | -2947 ^d^ | 5475 | -1457 ^f^ | 1.62 | -2283 (2853) | -2360 (3968) |
|  | Donepezil + Memantine | Donepezil alone | 52w | GBP (2013) | 25 | 547 ^d^ | -1204 | -331 ^f^ | 1.62 | 970 (2315) | -536 (3515) |
| Suh (2008) | Galantamine | No treatment | 52w | USD (2002) | 1465 | 1187 ^d^ | -7992 | -5372 ^f^ | 1.42 | 3766 (8034) | -7628 (2851) |
| Willan (2006) | Rivastigmine | Placebo | 24w | CAN (2004) | 722 | -107 ^d^ | -552 | 56 ^f^ | 1.11 | 799 (786) | 62 (2353) |
| Wimo (2003a) | Donepezil | Placebo | 52w | USD (1999) | 1280 | -1344 ^e^ | -1033 | -1097 | 1.50 | 437 (2233) | -1646 (3155) |
| Wimo (2003b) | Memantine | Placebo | 28w | USD (1999) | N.R | -1766 ^e^ | -5302 | -7014 | 1.50 | -1766 | -10562 (4247) |
| *Drugs aiming at psychological and behavioural symptoms of people with dementia* | | | | | | | | | | | |
| Banerjee (2013) | Mirtazapine | Sertraline | 39w | GBP (2009) | 30 | -319 | -1522 | 454 | 1.73 | -1019 (1694) | -3133 (2756) |
|  | Mirtazapine | Placebo | 39w | GBP (2009) | 37 | 367 | -1510 | -1811 | 1.73 | 180 (1673) | -1913 (2609) |
|  | Sertraline | Placebo | 39w | GBP (2009) | 7 | 686 | 12 | -1106 | 1.73 | 1199 (1692) | 1220 (3195) |
| Rosenheck (2007) | Olanzapine | Placebo | 36w | USD (2000) | 342 ^d^ | -252 | N.R | 90 | 1.47 | 106 (5610) | N.A |
|  | Risperidone | Placebo | 36w | USD (2000) | 423 ^d^ | -1962 | N.R | -1539 | 1.47 | -2276 (4944) | N.A |
|  | Quetiapine | Placebo | 36w | USD (2000) | 423 ^d^ | 1188 | N.R | 1611 | 1.47 | 2355 (7811) | N.A |
| *Study included in sensitivity analysis (Galantamine)* | | | | | | | | | | | |
| Wimo (2012) | Galantamine | Placebo | 28w | SEK  (2005) | N.R | N.R | N.R | -31035 | 0.15 | N.A | -4657 (3294) |

a. Cost data were presented as per participant or per dyad.

b. Incremental cost data extracted from studies were categorized into three types: cost of studied medications, cost of health and social care utilization, and cost incurred by informal care or productivity loss. The incremental total cost was a combination of them.

c. Conversion factors were derived from the CCEMG-EPPICentre Cost Converter (v.1.4) (link: <https://eppi.ioe.ac.uk/costconversion/>) and targeted at US dollar in 2020 price year.

d. Cost data were recalculated based on the formula that “total cost = program cost + healthcare utilization + informal or indirect cost”.

e. Cost collection included healthcare utilization of informal caregivers.

f. Adjusted values were reported in original studies, after controlling baseline characteristics or potential confounders.

*Notes: N.R, not reported; N.A, not available due to insufficient information*

##

## Table A10. Test of homogeneity on the costs and effects of drug therapies

(a) Costs based on mean differences and standardized mean differences

| Type of intervention | Incremental total cost (Intervention group – Control group) ^a, b^ | | | |
| --- | --- | --- | --- | --- |
|  | n | Healthcare perspective | n | Societal perspective |
| (01) Drugs for Alzheimer’s disease |  |  |  |  |
| Cholinesterase inhibitors | 5 | I^2^(MD): 0.57 (P=0.966)  I^2^(SMD): 0.60 (P=0.963) | 5 | I^2^(MD): 7.39 (P=0.117)  I^2^(SMD): 7.20 (P=0.126) |
| Memantine | 1 | n.a | 2 | I^2^(MD): 1.99 (P=0.158)  I^2^(SMD): 0.55 (P=0.459) |
| Propentofylline | 1 | n.a | 0 | n.a |
| Combination therapy | 1 | n.a | 1 | n.a |
| *(Test of differences between drug group)* |  | *I^2^(MD): 4.74 (P=0.190)*  *I^2^(SMD): 7.83 (P=0.050)^+^* |  | *I^2^(MD): 1.25 (P=0.530)*  *I^2^(SMD): 2.10 (P=0.350)* |
| (02) Drugs for neuropsychiatric symptoms of dementia |  |  |  |  |
| Antipsychotics | 3 | I^2^(MD): 0.27 (P=0.872)  I^2^(SMD): 0.30 (P=0.862) | 0 | n.a |
| Antidepressants | 2 | I^2^(MD): 0.18 (P=0.671)  I^2^(SMD): 0.61 (P=0.432) | 2 | I^2^(MD): 0.58 (P=0.457)  I^2^(SMD): 0.61 (P=0.439) |
| *(Test of differences between drug group)* |  | *I^2^(MD): 0.04 (P=0.840)*  *I^2^(SMD): 0.02 (P=0.880)* |  | n.a |

a. Negative values based on mean differences (MD) or standardized mean differences (SMD) indicated cost savings in the intervention group compared to the control group.

b. Test of homogeneity was based on Cochran's Q test. Values of I-square and corresponding P-value were presented, with a cut-off significance level of P=0.10.

*Notes: ^+^, P<0.10; n.a, not applicable*

(2) Effects based on standardized mean differences

| Type of intervention | Effects on health outcomes (Intervention group – Control group) ^a^ | | | | | | | | | | | |
| --- | --- | --- | --- | --- | --- | --- | --- | --- | --- | --- | --- | --- |
|  | n | Cognition | n | Activity functions | n | Global Deterioration | n | BPSD | n | Health Utility | n | QALY |
| (01) Drugs for Alzheimer’s disease |  |  |  |  |  |  |  |  |  |  |  |  |
| Cholinesterase inhibitors | 6 | I^2^: 21.17  (P<0.001)*^***^* | 6 | I^2^: 15.70  (P=0.008)*^**^* | 3 | I^2^: 2.22  (P=0.330) | 4 | I^2^: 7.65  (P=0.054)*^+^* | 1 | n.a | 2 | I^2^: 3.77  (P=0.052)*^+^* |
| Memantine | 2 | I^2^: 1.59  (P=0.207) | 2 | I^2^: 0.04  (P=0.846) | 1 | n.a | 1 | n.a | 1 | n.a | 1 | n.a |
| Propentofylline | 1 | n.a | 1 | n.a | 1 | n.a | 0 | n.a | 0 | n.a | 0 | n.a |
| Combination therapy | 1 | n.a | 1 | n.a | 0 | n.a | 1 | n.a | 1 | n.a | 1 | n.a |
| *(Test of differences between drugs)* |  | *I^2^: 6.53*  *(P=0.089)^+^* |  | *I^2^: 5.08*  *(P=0.166)* |  | *I^2^: 1.02*  *(P=0.601)* |  | *I^2^: 4.23*  *(P=0.121)* |  | *I^2^: 4.17*  *(P=0.124)* |  | *I^2^: 0.89*  *(P=0.640)* |
| (02) Drugs for neuropsychiatric symptoms of dementia |  |  |  |  |  |  |  |  |  |  |  |  |
| Antipsychotics | 0 | n.a | 3 | I^2^: 0.91  (P=0.634) | 3 | I^2^: 0.30  (P=0.861) | 0 | n.a | 3 | I^2^: 0.39  (P=0.824) | 3 | I^2^: 1.09  (P=0.581) |
| Antidepressants | 2 | I^2^: 1.40  (P=0.245) | 2 | I^2^: 0.05  (P=0.828) | 0 | n.a | 2 | I^2^: 0.68  (P=0.410) | 2 | I^2^: 0.10  (P=0.757) | 2 | I^2^: 0.34  (P=0.565) |
| *(Test of differences between drugs)* |  | *n.a* |  | *I^2^: 0.10*  *(P=0.750)* |  | *n.a* |  | *n.a* |  | *I^2^: 0.12*  *(P=0.718)* |  | *I^2^: 2.56*  *(P=0.104)* |

a. Positive values indicated better improvements in the health domain in the intervention group compared to the control group.

b. Test of homogeneity was based on Cochran's Q test. Values of I-square and corresponding P-value were presented, with a cut-off significance level of P=0.10.

*Notes: ^+^, P<0.10; ^*^, P<0.05; ^**^, P<0.01; ^***^, P<0.001; n.a, not applicable*

## Figure A4. Funnel plot and Egger’s test

| Acetylcholinesterase inhibitors^a^ | Funnel Plot  (Blue Dot = Studies, Grey Line = 95%CI, Red Line = Estimated β) | Egger’s test |
| --- | --- | --- |
| Incremental cost (healthcare perspective) | 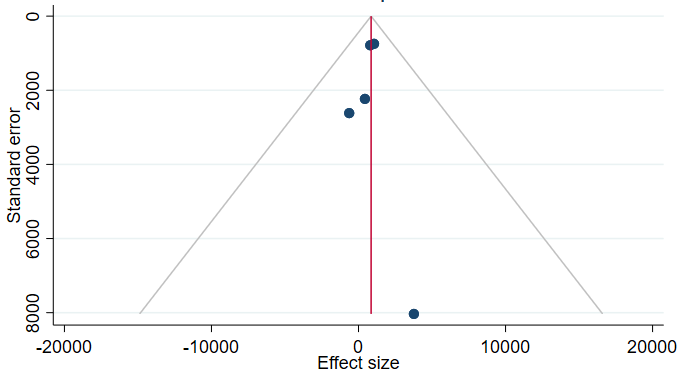 | n=5  β1(SE) = -0.13 (0.78)  z = -0.16, P= 0.870 |
| Incremental cost (societal perspective) | 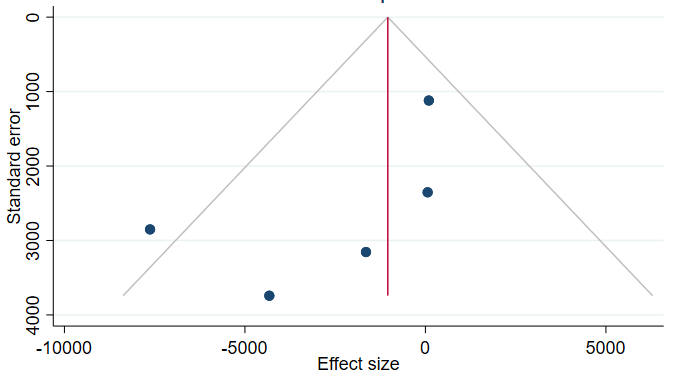 | n=5  β1(SE) = -1.91 (1.31)  z = -1.46, P= 0.144 |
| Cognitive function | 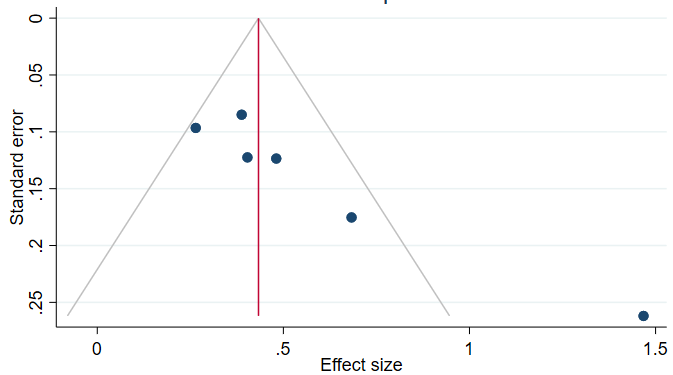  Knapp(2017) | n=6  β1(SE) = 5.48 (1.33)  z = 4.12, P < 0.000^b^ |
| Activity function | 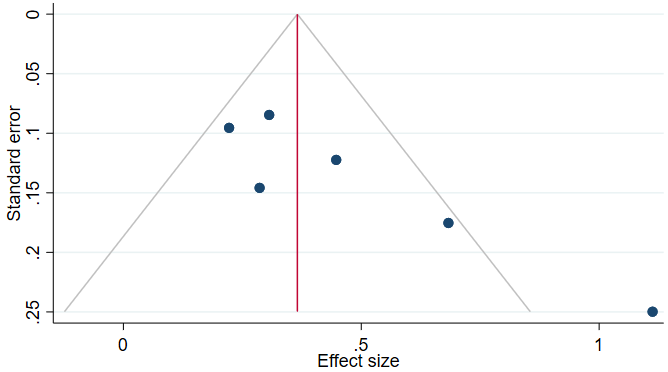  Knapp(2017) | n=6  β1(SE) = 4.33 (1.26)  z = 3.43, P < 0.000^b^ |
| Global deterioration | 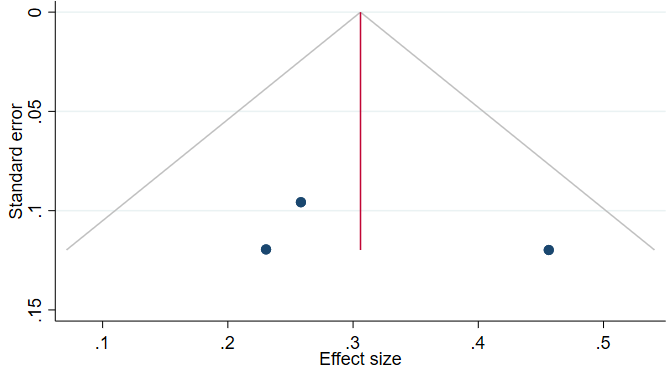 | n=3  β1(SE) = 3.62 (7.56)  z = 0.48, P= 0.632 |
| BPSD | 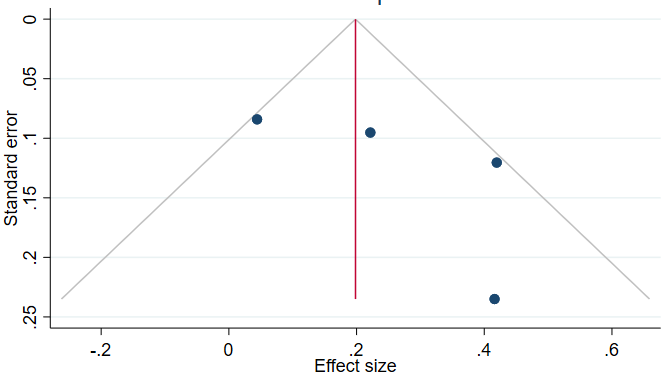 | n=4  β1(SE) = 2.43 (1.87)  z = 1.30, P= 0.194 |
| QALY | 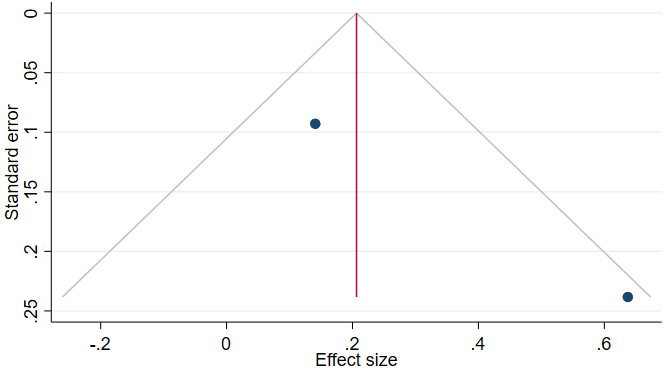  Knapp(2017) | n=2  β1(SE) = 3.41 (1.76)  z = 1.94, P= 0.052 |

a. Egger’s test was only performed for drugs of acetylcholinesterase inhibitors due to the number of included studies

b. P-value of Egger’s test reached a significant level lower than 0.05, which indicated small-study effects

## Figure A5. Cost-effectiveness plane of pharmacological interventions for people with dementia

(1) Drugs for Alzheimer’s disease

| (a) Cognition | (b) Activity functions |
| --- | --- |
| (c) Global deterioration | (d) BPSD |
| (c) Health Utility | (c) QALY |
| X-asix: Standardized Effect Size (Cohen’s d)  Y-axis: USD 2020 | Legends:  ○ Acetylcholinesterase inhibitors *vs* Placebo  ◇ Memantine *vs* Placebo  △ Propentofilline *vs* Placebo  ✕ Donepezil+Memantine *vs* Donepezil  Colors:  Orange: healthcare service perspective  Black: Sociteal perspective |

(2) Drug for neuropsychiatric symptoms of dementia

| (a) Cognition | (b) Activity functions |
| --- | --- |
| (c) Global deterioration | (d) BPSD |
| (c) Health Utility | (c) QALY |
| X-asix: Standardized Effect Size (Cohen’s d)  Y-axis: USD 2020 | Legends:  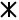 Antipsychotics  □ Antidepressants  Colors:  Orange: healthcare service perspective  Black: Sociteal perspective |

# Appendix 7: Subgroup analysis

## Table A11. Subgroup analysis of different perspectives on incremental total cost

| Type of intervention ^a^ | Incremental total cost (Intervention group – Control group) | | |
| --- | --- | --- | --- |
|  | n | Healthcare perspective | Societal perspective |
| Overall | 9 | MD: 411 (-660, 1483)  SMD: 0.047 (-0.069, 0.163) | MD: -2176 (-4140, -213) ^b^  SMD: -0.116 (-0.247, 0.015) |
| Drugs for Alzheimer’s disease | 6 | MD: 545 (-751, 1840)  SMD: 0.055 (-0.075, 0.185) | MD: -2627 (-5386, 132) ^b^  SMD: -0.139 (-0.317, 0.039) |
| - Acetylcholinesterase inhibitors | 4 | MD: 680 (-713, 2073)  SMD: 0.069 (-0.070, 0.209) | MD: -3150 (-6845, 545) ^b^  SMD: -0.182 (-0.438, 0.074) |
| - Memantine | 1 | MD: -2283 (-7874, 3309)  SMD: -0.213 (-0.736, 0.310) | MD: -2360 (-10100, 5417)  SMD: -0.158 (-0.681, 0.364) |
| - Combined use vs Monotherapy | 1 | MD: 970 (-3568, 5508)  SMD: 0.106 (-0.389, 0.600) | MD: -536 (-7426, 6353)  SMD: -0.038 (-0.532, 0.455) |
| Drugs for neuropsychiatric symptoms (antidepressants) | 2 | MD: 684 (-1648, 3015)  SMD: 0.091 (-0.222, 0.404) | MD: -660 (-4620, 3301)  SMD: -0.041 (-0.354, 0.272) |

a. Analysis was restricted to the five studies that reported costs from both healthcare service and societal perspective: studies of Knapp (2017), Suh (2008), Willan (2006), Wimo (2003a) and Banerjee (2013).

b. Regression meta-analysis indicated significant differences in incremental total cost between healthcare perspective and societal perspective (P<0.05) (Overall: difference in MD -2587, P=0.020; Drugs for Alzheimer’s disease: difference in MD -3172, P=0.025; Acetylcholinesterase inhibitors: -3830, P=0.038).

## Table A12. Subgroup analysis of including healthcare utilization of caregivers on incremental total cost

| Including healthcare utilization of caregivers or not | Incremental total cost (Intervention group – Control group) ^a^ | | | |
| --- | --- | --- | --- | --- |
|  | n | Healthcare perspective | n | Societal perspective |
| Acetylcholinesterase inhibitors |  |  |  |  |
| No | 5 | MD: 883 (-154, 1919)  SMD: 0.100 (-0.015, 0.215) | 3 | MD: -3704 (-8594, 1186)  SMD: -0.255 (-0.634, 0.124) |
| Yes | 1 | MD: 437 (-3939, 4812)  SMD: 0.028 (-0.255, 0.311) | 2 | MD: -99 (-2168, 1970)  SMD: -0.024 (-0.203, 0.155) |
| Memantine |  |  |  |  |
| No | 1 | MD: -2283 (-7874, 3309)  SMD: -0.213 (-0.736, 0.310) | 1 | MD: -2360 (-10100, 5417)  SMD: -0.158 (-0.681, 0.364) |
| Yes | 0 |  | 1 | MD: -6322 (-18900, -2239)  SMD: -0.387 (-0.696, -0.079) |

a. Regression meta-analysis indicated no significant differences in incremental cost between different subgroups (P>0.05).

## Table A13. Subgroup analysis based on baseline characteristics of people with dementia and social contexts (Acetylcholinesterase inhibitors)

(1) Incremental total cost

|  | Incremental total cost (Intervention group – Control group) | | | |
| --- | --- | --- | --- | --- |
|  | n | Healthcare perspective | n | Societal perspective |
| Follow-up periods |  |  |  |  |
| 24 weeks | 1 | MD: 799 (-742, 2340)  SMD: 0.094 (-0.088, 0.276) | 2 | MD: 90 (-1893, 2072)  SMD: 0.005 (-0.137, 0.148) |
| 52 weeks | 4 | MD: 904 (-431, 2238)  SMD: 0.088 (-0.043, 0.219) | 3 | MD: -4757 (-8595, -918) ^a^  SMD: -0.293 (-0.623, 0.037) |
| Severity |  |  |  |  |
| mild to moderate | 4 | MD: 919 (-110, 1947)  SMD: 0.096 (-0.013, 0.204) | 3 | MD: -2938 (-7613, 1738)  SMD: -0.165 (-0.468, 0.137) |
| moderate to severe | 1 | MD: -630 (-5758, 4498)  SMD: -0.073 (-0.664, 0.519) | 2 | MD: -672 (-3954, 2610)  SMD: -0.060 (-0.338, 0.219) |
| Mean age of sample |  |  |  |  |
| 70-74 years | 2 | MD: 759 (-694, 2212)  SMD: 0.075 (-0.078, 0.228) | 3 | MD: -72 (-1960, 1815)  SMD: -0.011 (-0.138, 0.116) |
| 75-79 years | 3 | MD: 952 (-449, 2353)  SMD: 0.104 (-0.044, 0.253) | 2 | MD: -6414 (-10900, -1969) ^a^  SMD: -0.492 (-0.836, -0.148) ^a^ |
| Social context^b^ |  |  |  |  |
| United Kingdom | 2 | MD: 929 (-477, 2336)  SMD: 0.105 (-0.054, 0.264) | 1 | MD: -4324 (-11700, 3010)  SMD: -0.349 (-0.945, 0.247) |
| South Korea | 1 | MD: 3766 (-12000, 19511)  SMD: 0.099 (-0.065, 0.226) | 1 | MD: -7628 (-13200, -2040)  SMD: -0.564 (-0.985, -0.143) |
| Multi-centres (including other European countries, Canada, Austria) | 2 | MD: 759 (-694, 2212)  SMD: 0.075 (-0.078, 0.228) | 3 | MD: -72 (-1960, 1815)  SMD: -0.011 (-0.138, 0.116) |
| Active component^c^ |  |  |  |  |
| Donepezil | 3 | MD: 883 (-456, 2222)  SMD: 0.087 (-0.052, 0.225) | 3 | MD: -410 (-2401, -1581)  SMD: -0.051 (-0.222, 0.120) |
| Galantamine | 1 | MD: 3766 (-12000, 19511)  SMD: 0.099 (-0.315, 0.512) | 1 | MD: -7628 (-13200, -2040)  SMD: -0.564 (-0.985, -0.143) |
| Rivastigmine | 1 | MD: 799 (-742, 2340)  SMD: 0.094 (-0.088, 0.276) | 1 | MD: 62 (-4550, 4674)  SMD: -0.564 (-0.179, 0.184) |

a. Regression meta-analysis indicated significant differences in the incremental cost of acetylcholinesterase inhibitors between different subgroups (P<0.05) (Follow-up periods: difference in MD of societal cost -4847, P=0.028; Mean age: difference in MD of societal cost -6342, P=0.010; Mean age: difference in MD of societal cost -0.481, P=0.015).

b. Comparisons were based on Unite Kingdom and other countries (South Korea, multi-centres).

c. Comparisons were based on donepezil and other drugs (galantamine, rivastigmine).

(2) Health outcomes

| Type of intervention | Effects on health outcomes (Intervention group – Control group) | | | | | | | | | | | |
| --- | --- | --- | --- | --- | --- | --- | --- | --- | --- | --- | --- | --- |
|  | n | Cognition | n | Activity functions | n | Global Deterioration | n | BPSD | n | Health Utility | n | QALY |
| Follow-up periods |  |  |  |  |  |  |  |  |  |  |  |  |
| 24 weeks | 2 | 0.359  (0.149, 0.569) | 2 | 0.322  (0.103, 0.541) | 2 | 0.344  (0.152, 0.536) | 2 | 0.307  (0.115, 0.499) | 0 |  | 1 | 0.141  (-0.041, 0.323) |
| 52 weeks | 4 | 0.691  (0.241, 1.142) | 4 | 0.550  (0.204, 0.896) | 1 | 0.231  (-0.004, 0.465) | 2 | 0.165  (-0.176, 0.507) | 1 | -0.323  (-0.781, 0.136) | 1 | 0.637  (0.170, 1.104) |
| Severity |  |  |  |  |  |  |  |  |  |  |  |  |
| mild to moderate | 4 | 0.383  (0.271, 0.496) | 4 | 0.325  (0.190, 0.460) | 2 | 0.247  (0.101, 0.394) | 2 | 0.127  (-0.046, 0.301) | 0 |  | 1 | 0.141  (-0.041, 0.323) |
| moderate to severe | 2 | 0.947  (-0.018, 1.913) | 2 | 0.744  (0.096, 1.392) | 1 | 0.456  (0.221, 0.691) | 2 | 0.419 ^a^  (0.209, 0.629) | 1 | -0.323  (-0.781, 0.136) | 1 | 0.637  (0.170, 1.104) |
| Mean age of sample |  |  |  |  |  |  |  |  |  |  |  |  |
| 70-74 years | 3 | 0.365  (0.230, 0.500) | 3 | 0.306  (0.162, 0.450) | 0 |  | 2 | 0.307  (0.115, 0.499) | 0 |  | 1 | 0.141  (-0.041, 0.323) |
| 75-79 years | 3 | 0.808  (0.200, 1.416) | 3 | 0.655  (0.206, 1.104) | 3 | 0.306  (0.181, 0.432) | 2 | 0.165  (-0.176, 0.507) | 1 | -0.323  (-0.781, 0.136) | 1 | 0.637  (0.170, 1.104) |
| Social context^b^ |  |  |  |  |  |  |  |  |  |  |  |  |
| UK | 2 | 0.899  (-0.157, 1.956) | 2 | 0.675  (-0.112, 1.462) | 0 |  | 2 | 0.165  (-0.176, 0.507) | 1 | -0.323  (-0.781, 0.136) | 1 | 0.637  (0.170, 1.104) |
| South Korea | 1 | 0.683  (0.340, 1.027) | 1 | 0.683  (0.340, 1.027) | 0 |  | 0 |  | 0 |  | 0 |  |
| Multi-centres | 3 | 0.365  (0.230, 0.500) | 3 | 0.306  (0.162, 0.450) | 3 | 0.306  (0.181, 0.432) | 2 | 0.307  (0.115, 0.499) | 0 |  | 1 | 0.141  (-0.041, 0.323) |
| Active component^c^ |  |  |  |  |  |  |  |  |  |  |  |  |
| donepezil | 4 | 0.639  (0.189, 1.089) | 4 | 0.482  (0.180, 0.784) | 2 | 0.343  (0.122, 0.564) | 3 | 0.260  (-0.018, 0.537) | 1 | -0.323  (-0.781, 0.136) | 1 | 0.637  (0.170, 1.104) |
| galantamine | 1 | 0.683  (0.340, 1.027) | 1 | 0.683  (0.340, 1.027) | 0 |  | 0 |  | 0 |  | 0 |  |
| rivastigmine | 1 | 0.265  (0.075, 0.454) | 1 | 0.222  (0.035, 0.409) | 1 | 0.258  (0.071, 0.446) | 1 | 0.222  (0.035, 0.408) | 0 |  | 1 | 0.141  (-0.041, 0.323) |

a. Regression meta-analysis indicated significant differences in the incremental effectiveness of acetylcholinesterase inhibitors between different subgroups (P<0.05) (Severity: difference in SMD of BPSD 0.292, P=0.036).

b. Comparisons were based on Unite Kingdom and other countries (South Korea, multi-centres).

c. Comparisons were based on donepezil and other drugs (galantamine, rivastigmine).

# Appendix 8: Sensitivity analysis

## Table A14. Sensitivity analyses based on RCTs (n=9)

(1) Meta-analysis on incremental cost

| Type of intervention | Incremental total cost (Intervention group – Control group) ^a,b^ | | | |
| --- | --- | --- | --- | --- |
|  | n | Healthcare perspective | n | Societal perspective |
| (01) Drugs for Alzheimer’s disease |  |  |  |  |
| Cholinesterase inhibitors | 4 | MD: 847 (-164, 1858)  SMD: 0.090 (-0.021, 0.200) | 4 | MD: -336 (-2164, 1492)  SMD: -0.026 (-0.150, 0.099) |
| Memantine | 1 | N.D | 2 | N.D |
| Propentofylline | 1 | N.D | 0 | N.D |
| Combination therapy | 1 | N.D | 1 | N.D |
| (02) Drugs for neuropsychiatric symptoms of dementia |  |  |  |  |
| Antipsychotics | 3 | N.D | 0 | N.D |
| Antidepressants | 3 | N.D | 3 | N.D |

a. Negative values based on mean differences (MD) or standardized mean differences (SMD) indicated cost savings in the intervention group compared to the control group.

b. One study (Suh, 2008) was excluded due to non-randomized trial design.

*Notes: ^*^, P-value<0.05; ^**^, P-value<0.01; ^***^, P-value<0.001; N.D: no difference was found between sensitivity and primary analysis*

(2) Meta-analysis on effects of pharmacological interventions for people with dementia

| Type of intervention | Effects on health outcomes (Intervention group – Control group) ^a,b^ | | | | | | | | | | | |
| --- | --- | --- | --- | --- | --- | --- | --- | --- | --- | --- | --- | --- |
|  | n | Cognition | n | Activity functions | n | Global Deterioration | n | BPSD | n | Health Utility | n | QALY |
| (01) Drugs for Alzheimer’s disease |  |  |  |  |  |  |  |  |  |  |  |  |
| Cholinesterase inhibitors | 5 | 0.551^**^  (0.195, 0.907) | 6 | 0.408^***^  (0.187, 0.630) | 3 | N.D | 4 | N.D | 1 | N.D | 2 | N.D |
| Memantine | 2 | N.D | 2 | N.D | 1 | N.D | 1 | N.D | 1 | N.D | 1 | N.D |
| Propentofylline | 1 | N.D | 1 | N.D | 1 | N.D | 0 | N.D | 0 | N.D | 0 | N.D |
| Combination therapy | 1 | N.D | 1 | N.D | 0 | N.D | 1 | N.D | 1 | N.D | 1 | N.D |
| (02) Drugs for neuropsychiatric symptoms |  |  |  |  |  |  |  |  |  |  |  |  |
| Antipsychotics | 0 | N.D | 3 | N.D | 3 | N.D | 0 | N.D | 3 | N.D | 3 | N.D |
| Antidepressants | 3 | N.D | 3 | N.D | 0 | N.D | 3 | N.D | 3 | N.D | 3 | N.D |

a. Positive values indicated better improvements in the health domain in the intervention group compared to the control group.

b. One study (Suh, 2008) was excluded due to non-randomized trial design.

*Notes: ^*^, P-value<0.05; ^**^, P-value<0.01; ^***^, P-value<0.001; N.D: no difference was found between sensitivity and primary analysis*

## Table A15. Sensitivity analyses based on industry-sponsored studies

(1) Meta-analysis on incremental cost

| Type of intervention | Incremental total cost (Intervention group – Control group) ^a,b^ | | | |
| --- | --- | --- | --- | --- |
|  | n | Healthcare perspective | n | Societal perspective |
| (01) Drugs for Alzheimer’s disease |  |  |  |  |
| Cholinesterase inhibitors | 3 | MD: 784 (-663, 2231)  SMD: 0.078 (-0.066, 0.221) | 4 | MD: -2002 (-4944, 939)  SMD: -0.089 (-0.265, 0.087) |
| Memantine | 0 |  | 0 | N.D |
| Propentofylline | 1 | N.D | 0 | N.D |
| Combination therapy | 0 |  | 0 |  |
| (02) Drugs for neuropsychiatric symptoms of dementia |  |  |  |  |
| Antipsychotics | 3 | N.D | 0 | N.D |
| Antidepressants | 3 | N.D | 3 | N.D |

a. Negative values based on mean differences (MD) or standardized mean differences (SMD) indicated cost savings in the intervention group compared to the control group.

b. Three studies (Wimo, 2003b; Courtney, 2004; Knapp, 2017) were excluded due to non-industrial sponsorships.

*Notes: ^*^, P-value<0.05; ^**^, P-value<0.01; ^***^, P-value<0.001; N.D: no difference was found between sensitivity and primary analysis*

(2) Meta-analysis on effects of pharmacological interventions for people with dementia

| Type of intervention | Effects on health outcomes (Intervention group – Control group) ^a,b^ | | | | | | | | | | | |
| --- | --- | --- | --- | --- | --- | --- | --- | --- | --- | --- | --- | --- |
|  | n | Cognition | n | Activity functions | n | Global Deterioration | n | BPSD | n | Health Utility | n | QALY |
| (01) Drugs for Alzheimer’s disease |  |  |  |  |  |  |  |  |  |  |  |  |
| Cholinesterase inhibitors | 4 | 0.421^***^  (0.265, 0.577) | 4 | 0.380^***^  (0.197, 0.563) | 3 | N.D | 2 | 0.307^**^  (0.115, 0.499) | 0 |  | 1^b^ | 0.141  (-0.041, 0.323) |
| Memantine | 0 |  | 0 |  | 0 |  | 0 |  | 0 |  | 0 |  |
| Propentofylline | 1 | N.D | 1 | N.D | 1 | N.D | 0 | N.D | 0 | N.D | 0 | N.D |
| Combination therapy | 0 |  | 0 |  | 0 |  | 0 |  | 0 |  | 0 |  |
| (02) Drugs for neuropsychiatric symptoms |  |  |  |  |  |  |  |  |  |  |  |  |
| Antipsychotics | 0 | N.D | 3 | N.D | 3 | N.D | 0 | N.D | 3 | N.D | 3 | N.D |
| Antidepressants | 3 | N.D | 3 | N.D | 0 | N.D | 3 | N.D | 3 | N.D | 3 | N.D |

a. Positive values indicated better improvements in the health domain in the intervention group compared to the control group.

b. Three studies (Wimo, 2003b; Courtney, 2004; Knapp, 2017) were excluded due to non-industrial sponsorships.

*Notes: ^*^, P-value<0.05; ^**^, P-value<0.01; ^***^, P-value<0.001; N.D: no difference was found between sensitivity and primary analysis*

## Table A16. Comparisons of self-rated scales and proxy-rated scales on health-related quality of life of people with dementia

| Type of intervention | n | Self-rated  HRQoL | n | Proxy-rated  HRQoL | n | Self-rated  QALY | n | Proxy-rated  QALY |
| --- | --- | --- | --- | --- | --- | --- | --- | --- |
| (01) Drugs for Alzheimer’s disease |  |  |  |  |  |  |  |  |
| Cholinesterase inhibitors | 0 |  | 1 | -0.323  (-0.781, 0.136) | 2 | 0.341  (-0.136, 0.818) | 0 |  |
| Memantine | 0 |  | 1 | 0.224  (-0.170, 0.618) | 1 | 0.327  (-0.068, 0.772) | 0 |  |
| Propentofylline | 0 |  | 0 |  | 0 |  | 0 |  |
| Combination therapy | 0 |  | 1 | 0.249  (-0.148, 0.646) | 1 | 0.092  (-0.303, 0.488) | 0 |  |
| (02) Drugs for neuropsychiatric symptoms of dementia |  |  |  |  |  |  |  |  |
| Antipsychotics | 3 | -0.022  (-0.225, 0.181) | 0 |  | 3 | 0.020  (-0.183, 0.223) | 0 |  |
| Antidepressants | 2 | -0.146  (-0.520, 0.227) | 2 | 0.159  (-0.214, 0.533) | 2 | 0.378  (0.002, 0.754) | 0 |  |

a. Positive values indicated better improvements in the health domain in the intervention group compared to the control group.

*Notes: ^*^, P-value<0.05; ^**^, P-value<0.01; ^***^, P-value<0.001; HRQoL, health-related quality of life; N.D: no difference was found between sensitivity and primary analysis; QALY, quality adjusted life year*

## Table A17. Sensitivity analyses based on studies with complete data on both costs and effects

(1) Cost in relationship to Cognition, ADL, Global deterioration

| Type of intervention |  | Effects on total cost (MD) ^a^ and caregiver health outcomes (SMD) ^b^ (Intervention group – Control group) | | | | | | | | |
| --- | --- | --- | --- | --- | --- | --- | --- | --- | --- | --- |
|  | Perspective | n | Incremental Cost | Cognitive functions | n | Incremental Cost | Activity functions | n | Incremental Cost | Global deterioration |
| (01) Drugs for Alzheimer’s disease | | | | | | | | | | |
| Cholinesterase inhibitors | Healthcare | 5 | 1431^*^  (68, 2795) | 0.594^*^  (0.223, 0.965) | 5 | 1431^*^  (68, 2795) | 0.469^**^  (0.188, 0.750) | 2 | 759  (-694, 2212) | 0.247^***^  (0.101, 0.394) |
|  | Societal | 5 | -2602  (-6418, 1214) | 0.614^**^  (0.249, 0.978) | 5 | -2602  (-6418, 1214) | 0.501^***^  (0.227, 0.774) | 3 | -72  (-1960, 1815) | 0.306^***^  (0.181, 0.432) |
| Memantine | Healthcare | 1^c^ |  |  | 1^c^ |  |  | 0 |  |  |
|  | Societal | 2 | -6322  (-14355, 1711) | 0.602^***^  (0.290, 0.914) | 2 | -6322  (-14355, 1711) | 0.445^***^  (0.208, 0.682) | 1^c^ |  |  |
| Propentofylline | Healthcare | 1^c^ |  |  | 1^c^ |  |  | 1^c^ |  |  |
|  | Societal | 0 |  |  | 0 |  |  | 0 |  |  |
| Combined therapy | Healthcare | 1^c^ |  |  | 1^c^ |  |  | 0 |  |  |
|  | Societal | 1^c^ |  |  | 1^c^ |  |  | 0 |  |  |
| (02) Drugs for neuropsychiatric symptoms | | | | | | | | | | |
| Antipsychotics | Healthcare | 0 |  |  | 3 | -574  (-7141, 5993) | -0.192^*^  (-0.369, -0.014) | 3 | -574  (-7141, 5993) | 0.189  (-0.014, 0.392) |
|  | Societal | 0 |  |  | 0 |  |  | 0 |  |  |
| Antidepressants | Healthcare | 3 | 122  (-1787, 2030) | -0.328^*^  (-0.592, -0.065) | 3 | 122  (-1787, 2030) | -0.140  (-0.402, 0.122) | 0 |  |  |
|  | Societal | 3 | -1524  (-4719, 1670) | -0.328^*^  (-0.592, -0.065) | 3 | -1524  (-4719, 1670) | -0.140  (-0.402, 0.122) | 0 |  |  |

(2) Cost in relationship to BPSD, QoL, QALY

| Type of intervention |  | Effects on total cost (MD) ^a^ and caregiver health outcomes (SMD) ^b^ (Intervention group – Control group) | | | | | | | | |
| --- | --- | --- | --- | --- | --- | --- | --- | --- | --- | --- |
|  | Perspective | n | Incremental Cost | BPSD | n | Incremental Cost | Health Utility | n | Incremental Cost | QALY |
| (01) Drugs for Alzheimer’s disease | | | | | | | | | | |
| Cholinesterase inhibitors | Healthcare | 3 | 870  (-169, 1909) | 0.162  (-0.009, 0.333) | 1^c^ |  |  | 2 | 681  (-795, 2156) | 0.341  (-0.136, 0.818) |
|  | Societal | 3 | -211  (-2124, 1703) | 0.317^***^  (0.155, 0.478) | 1^c^ |  |  | 2 | -1181  (-5085, 2723) | 0.341  (-0.136, 0.818) |
| Memantine | Healthcare | 1^c^ |  |  | 1^c^ |  |  | 1^c^ |  |  |
|  | Societal | 1^c^ |  |  | 1^c^ |  |  | 1^c^ |  |  |
| Propentofylline | Healthcare | 0 |  |  | 0 |  |  | 0 |  |  |
|  | Societal | 0 |  |  | 0 |  |  | 0 |  |  |
| Combined therapy | Healthcare | 1^c^ |  |  | 1^c^ |  |  | 1^c^ |  |  |
|  | Societal | 1^c^ |  |  | 1^c^ |  |  | 1^c^ |  |  |
| (02) Drugs for neuropsychiatric symptoms | | | | | | | | | | |
| Antipsychotics | Healthcare | 0 |  |  | 3 | -574  (-7141, 5993) | -0.022  (-0.225, 0.181) | 3 | -574  (-7141, 5993) | 0.020  (-0.183, 0.223) |
|  | Societal | 0 |  |  | 0 |  |  | 0 |  |  |
| Antidepressants | Healthcare | 3 | 122  (-1787, 2030) | 0.110  (-0.150, 0.369) | 3 | 122  (-1787, 2030) | 0.043  (-0.264, 0.350) | 3 | 122  (-1787, 2030) | 0.327^*^  (0.018, 0.635) |
|  | Societal | 3 | -1524  (-4719, 1670) | 0.110  (-0.150, 0.369) | 3 | -1524  (-4719, 1670) | 0.043  (-0.264, 0.350) | 3 | -1524  (-4719, 1670) | 0.327^*^  (0.018, 0.635) |

a. Negative values based on mean differences (MDs) indicated cost savings in the intervention group compared to the control group.

b. Positive values based on standardized mean differences (SMD) indicated better improvements in the health domain in the intervention group compared to the control group.

c. Meta-synthesis was not performed due to insufficient number of studies (n=1).

*Notes: ^*^, P-value<0.05; ^**^, P-value<0.01; ^***^, P-value<0.00*

**Reference (Following full text)**

41. Kittner B, Rössner M, Rother M. Clinical Trials in Dementia with Propentofylline a. Ann N sY Acad Sci. 1997;826(1):307-16.

42. Rother M, Erkinjuntti T, Roessner M, Kittner B, Marcusson J, Karlsson I. Propentofylline in the treatment of Alzheimer’s disease and vascular dementia: a review of phase III trials. Dement Geriatr Cogn Disord. 1998;9(Suppl. 1):36-43.

43. Rother M, Kittner B, Rudolphi K, Rössner M, LABS KH. HWA 285 (propentofylline)—a new compound for the treatment of both vascular dementia and dementia of the Alzheimer type. Ann N Y Acad Sci. 1996;777(1):404-9.

44. Feldman H, Gauthier S, Hecker J, Vellas B, Emir B, Mastey V, et al. Efficacy of donepezil on maintenance of activities of daily living in patients with moderate to severe Alzheimer's disease and the effect on caregiver burden. J Am Geriatr Soc. 2003;51(6):737-44.

45. Feldman H, Gauthier S, Hecker J, Vellas B, Subbiah P, Whalen E. A 24-week, randomized, double-blind study of donepezil in moderate to severe Alzheimer’s disease. Neurology. 2001;57(4):613-20.

46. Gauthier S, Feldman H, Hecker J, Vellas B, Ames D, Subbiah P, et al. Efficacy of donepezil on behavioral symptoms in patients with moderate to severe Alzheimer's disease. Int Psychogeriatr. 2002;14(4):389-404.

47. Howard R, McShane R, Lindesay J, Ritchie C, Baldwin A, Barber R, et al. Donepezil and memantine for moderate-to-severe Alzheimer's disease. N Engl J Med. 2012;366(10):893-903.

48. Suh G-H, Jung HY, Lee CU, Oh BH, Bae JN, Jung H-Y, et al. A prospective, double-blind, community-controlled comparison of three doses of galantamine in the treatment of mild to moderate Alzheimer's disease in a Korean population. Clin Ther. 2004;26(10):1608-18.

49. Emre M, Aarsland D, Albanese A, Byrne EJ, Deuschl G, De Deyn PP, et al. Rivastigmine for dementia associated with Parkinson's disease. N Engl J Med. 2004;351(24):2509-18.

50. Reisberg B, Windscheif U, Ferris SH, Hingorani VN, Stoeffler A, Moebius H-J. Memantine in moderately severe to severe Alzheimer's disease (AD): results of a placebo-controlled 6-month trial. Neurobiol Aging. 2000(21):275.

51. Schneider LS, Tariot PN, Dagerman KS, Davis SM, Hsiao JK, Ismail MS, et al. Effectiveness of atypical antipsychotic drugs in patients with Alzheimer's disease. N Engl J Med. 2006;355(15):1525-38.

52. Wilcock GK, Lilienfeld S, Gaens E. Efficacy and safety of galantamine in patients with mild to moderate Alzheimer's disease: multicentre randomised controlled trial. BMJ. 2000;321(7274):1445.
